# Modifiable predictors of health literacy in working-age adults - a rapid review and meta-analysis

**DOI:** 10.1186/s12889-022-13851-0

**Published:** 2022-07-30

**Authors:** Hunny Singh, Jonathan Kolschen, Florence Samkange-Zeeb, Tilman Brand, Hajo Zeeb, Benjamin Schüz

**Affiliations:** 1 Institute of Public Health and Nursing Research, University of Bremen, Bremen, Germany; 2 Department of Prevention and Evaluation, Leibniz Institute for Prevention Research and Epidemiology – BIPS, Bremen, Germany; 3 Health Sciences Bremen, University of Bremen, Bremen, Germany

**Keywords:** Health literacy, Long-term unemployment, Rapid review, Meta-analysis, Language proficiency, Internet use

## Abstract

**Background:**

Health literacy comprises the ability to identify, obtain, interpret and act upon health information. Low health literacy is a major risk factor for hospitalizations, use of emergency care and premature mortality among others. Known risk factors for low health literacy such as lower educational attainment, migration history and chronic illnesses overlap with those for long-term unemployment – in itself a risk factor for low health literacy. These factors are difficult to address in interventions to support health literacy. Therefore, the objective of this review is to identify potentially modifiable predictors of HL in populations potentially affected by long-term unemployment.

**Methods:**

A rapid review (PROSPERO registration number: 290873) was carried out in Pubmed and SCOPUS including quantitative studies on potentially modifiable predictors of health literacy in working-age populations following PRISMA guidelines for systematic reviews. Where possible, reported effect sizes were transformed into r, and random-effects meta-analyses were conducted where appropriate to pool effect sizes for the association between modifiable predictors and health literacy.

**Results:**

In total, 4765 titles and abstracts were screened, 114 articles were assessed in full-text screening, and 54 were included in the review. Forty-one effect sizes were considered for 9 different meta-analyses. Higher language proficiency, higher frequency of internet use, using the internet as a source of health information more often, being more physically active, more oral health behaviours, watching more health-related TV and a good health status were significantly associated with higher health literacy. Significant heterogeneity suggests between-study differences.

**Conclusions:**

Improving language proficiency and/or providing information in multiple and simplified languages, together with reliable and accessible health information on the internet and in linear media are potentially promising targets to improve health literacy levels in working-age populations.

**Supplementary Information:**

The online version contains supplementary material available at 10.1186/s12889-022-13851-0.

## Introduction

The World Health Organization defines health literacy (HL) as “knowledge, personal skills, and confidence to take action to improve personal and community health by changing personal lifestyles and living conditions” [1]. Over the years, more definitions of HL were developed, and the European Health Literacy Consortium analysed 17 HL definitions and summarized the essential aspects of them as: “… people’s knowledge, motivation and competences to access, understand, appraise and apply health information in order to make judgements and take decisions in everyday life concerning healthcare, disease prevention and health promotion” [2].

Lower HL has been identified as a major risk factor for adverse health or health behaviour outcomes such as more hospitalizations, higher emergency care use, higher mortality rates, poorer self-care management, lower medication adherence, lower participation in screening programs or lower levels of preventive behaviours [3–9].

A recent study showed that compared to people with adequate HL, those with inadequate HL had poorer understanding of COVID-19 symptoms, were less able to identify behaviours to prevent infection, and struggled more to find information and understand government messaging about COVID-19 [10]. Unfortunately, low levels of HL are highly prevalent; a European survey indicated insufficient or limited HL among 59% of the participants [11]. Comparable results were found in a survey in Germany [12] and in a systematic review summarizing HL studies in Asian countries [13]. Certain population groups, such as people with lower socioeconomic status, lower educational attainment, older adults, people with a migration background, or who have been unemployed for a long time, are at particularly high risk of low HL [11, 12, 14].

More often than not, risk factors for low HL overlap with indicators for social deprivation. In Germany, lower HL is prevalent in people without formal professional qualification, persons aged 55 and above, persons with a migration history who are not fluent in the majority language of the country, as well as those with chronic mental or physical illnesses – those who are also most at risk for long-term unemployment [15]. This indicates a reciprocal relationship between unemployment and illness, i.e. that chronic illness does not only increase the risk for unemployment, but that unemployment itself can also increase the risk of low health literacy and subsequently ill health [16]. Known risk factors for lower HL mentioned above mainly represent sociodemographic characteristics of individuals or groups, which mainly overlap with the known social determinants of health. Addressing these risk factors requires co-ordinated system-based upstream health promotion interventions, which may need complementing with downstream interventions that target modifiable risk factors for low health literacy – both in the general population as well as in high-risk populations such as long-term unemployed individuals. Therefore, more needs to be known about factors that can be modified more easily and hence be addressed in interventions.

The main aim of this rapid review is to summarize and meta-analyse the literature on potentially modifiable predictors of HL among working-age populations. Importantly, this requires focusing on primary studies that have examined HL as an outcome of potentially modifiable factors.

This review was conducted as part of the FORESIGHT project, in which we develop a framework for evidence-based interventions to promote HL in an occupational rehabilitation setting [17].

## Methods

We conducted a rapid review on modifiable downstream predictors of HL and followed the PRISMA guidelines for systematic reviews. A modifiable predictor was considered to be an individual-level feature or a behaviour that is susceptible to change through either broad-based individual choices or public policy choices [18]. This includes health behaviours and provision of information sources or materials/tools needed to promote HL, but excludes sociodemographic and socioeconomic indicators, which require upstream interventions. The protocol for this review was registered in November 2021 at PROSPERO (registration number: 290873).

### Eligibility criteria and information sources

To identify relevant articles for the rapid review, we searched the databases Pubmed (via Ovid) and SCOPUS (via Elsevier) in August 2021 from 1998 onwards. Pubmed covers the widest range of journal articles in biomedical research and SCOPUS additionally covers selections from the social sciences, basic sciences and humanities.

#### Inclusion criteria

Primary studies published from 1998 onwards^1^ with a quantitative study design, investigating potentially modifiable predictors of HL in populations between 18 and 65 years of age.^2^ We chose this age range as we focused on populations that could potentially be long-term unemployed, in other words – working-age populations.

#### Exclusion criteria

Articles not reporting HL as an outcome, not reporting some kind of effect estimators or statistical tests, not published in English or German, and where the full-texts were not accessible.

##### Search strategy

The search terms were “predictors”, “determinants”, “association”, “correlation”, “relationship” and “health literacy”. Search results were limited to adult populations by excluding the terms “children” and “adolescents”. Populations with a mean age over 65 years were excluded during the title/abstract and full-text screening. Websites or relevant national and international institutions were searched to identify grey literature. Additionally, references of the included articles were searched for further relevant literature. The title and abstract screening was done by one author (HS) and full-text screening by two authors (HS & JK). Interrater reliability for full text inclusion was satisfactory (Cohen’s Kappa = 0.75, percentage agreement = 87.71%). Data extraction was divided between two authors (HS & JK), with extracted data checked against the publication by the respective other author. Conflicts were resolved by discussion until consensus was reached.

### Data charting

Findings were summarized in a data extraction table (see Additional file 1) including bibliographic information of the study, study design, HL instruments, modifiable predictors of HL, and main findings. The risk of bias of individual studies was assessed using the NIH (National Institutes of Health) Quality Assessment Tool [19].

### Meta-analysis

All effect sizes of bivariate associations reported in the studies (e.g., correlations, Odds Ratios, t values, F values, eta squared) were converted to Pearson’s r. If studies reported both multivariate and bivariate associations, only bivariate associations were extracted. Results from studies that reported multivariate analyses only were not considered for meta-analyses but were included in narrative analyses. Details of all extracted data can be found in Additional file 1. Random-effects meta-analyses were conducted for those potentially modifiable predictors that were reported in more than two studies to identify the strength of the association between modifiable predictors and HL as well as the heterogeneity in these associations. All meta-analyses were performed with Jamovi (version1.6.23.0). Additional file 2 shows all converted effect sizes.

### Sensitivity analyses

Sensitivity analyses were conducted for articles that did not specifically report the mean age or age range of the study populations. Most of the articles concerned reported age group distributions of the study population. To also include studies without this information, we separately analysed them and compared their findings to the main results (see Additional file 3).

## Results

**Fig. 1:**
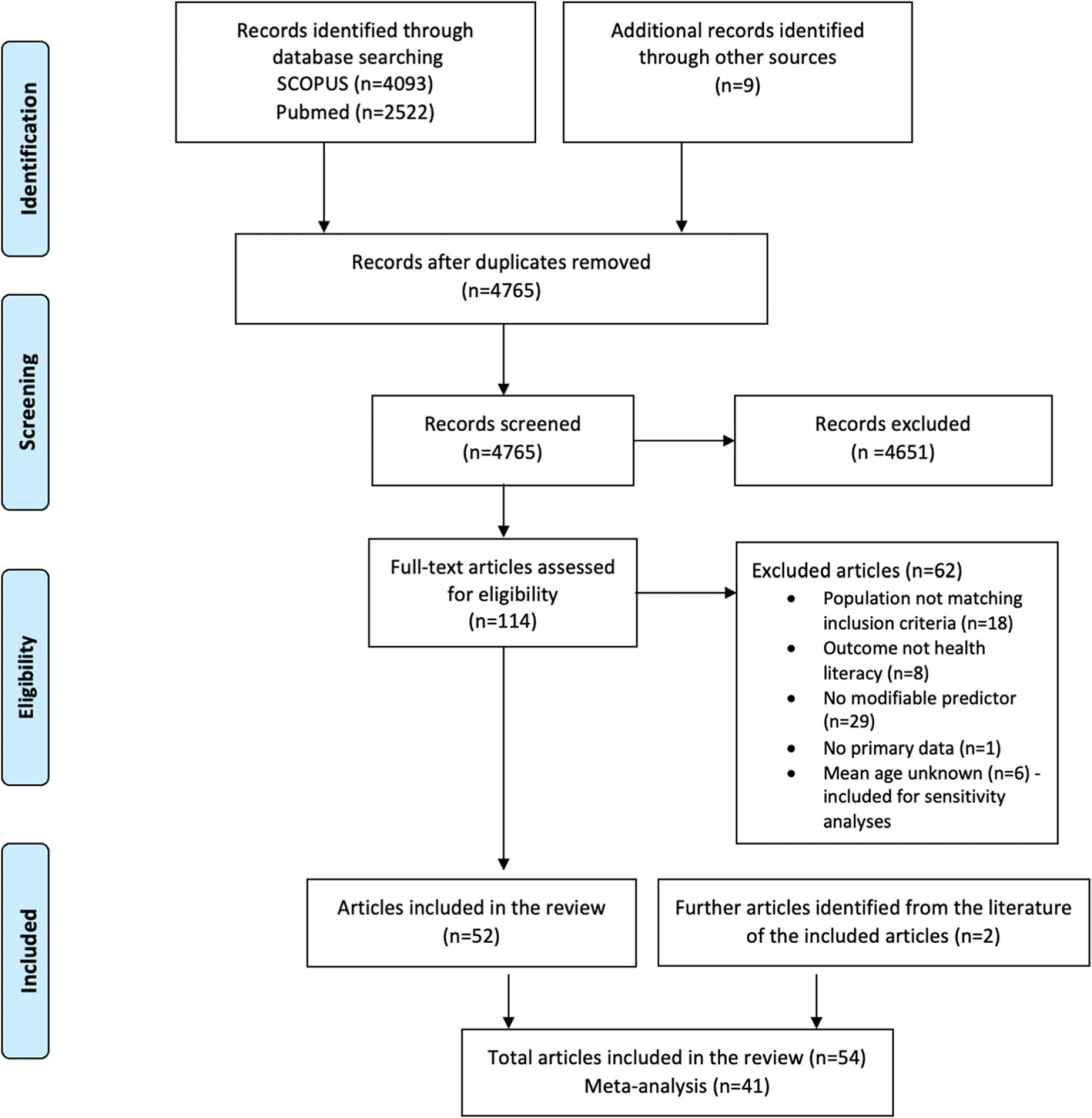
PRISMA flow chart of the search process

From the 4765 titles and abstracts screened, 114 articles were included in the full-text phase. Fifty-two articles subsequently met the eligibility criteria and were included in the rapid review. Two further papers were added based on hand-search of reference lists of the included articles, bringing the total number of articles analysed to 54 (Fig. 1).

Main reasons for exclusion during the full-text screening were not reporting modifiable predictors (*n* = 29), the study population not matching inclusion criteria (*n* = 18) and not assessing HL as an outcome but as an exposure for another outcome (*n* = 8). Six further articles that did not provide the mean age of the study population were not included in the main analyses, but in sensitivity analyses (see Additional file 3).

### Study characteristics

The 54 studies included in the review were conducted in 26 different countries, with the majority being conducted in the USA (*n* = 11) [20–30], in Iran (*n* = 8) [31–38], in Turkey (*n* = 5) [39–43] and in Australia (*n* = 3) [44–46].

With one exception (cohort study), all included studies had a cross-sectional (*n* = 53) design. The sample sizes of the studies ranged from *n* = 75 to *n* = 8362 and the average sample size was *n* = 818. All studies were written in English and published between 2009 and 2021.

The study populations were heterogeneous and included university, nursing and college students [26, 33, 36, 41, 47–51], teachers (including preschool, primary and secondary class teachers) [43, 52], clinical populations/patients attending healthcare clinics [25, 29, 44, 45, 53–58], immigrant populations [20, 21, 28], general adult populations (according to the place where the study was conducted) [35, 37–39, 46, 59–64] and prisoners [65] (see Additional file 4 for HL levels in these different populations).

Nevertheless, the objectives were similar across included articles. Two main aims could be identified: (a) depicting HL levels of the population under investigation and (b) assessing predictors and associated factors of HL. Further aims included the validation of HL survey tools, oral HL tools and e-HL scales.

### Health literacy outcomes and instruments

Studies assessed general HL (*n* = 34), e-health literacy (*n* = 9), oral health literacy (*n* = 8) and mental health literacy (*n* = 2). The instruments employed most often were the HLS-EU-Q47 [61, 65, 66], the HLS-EU-Q16 [40, 67], the Newest Vital Sign (NVS) [23, 28, 29, 43, 60], the HLS-SF12 [64, 68] and the S-TOFHLA [25, 30, 53]. All tools are given in the data extraction table (see Additional file 1).

### Risk of bias analysis

**Fig. 2:**
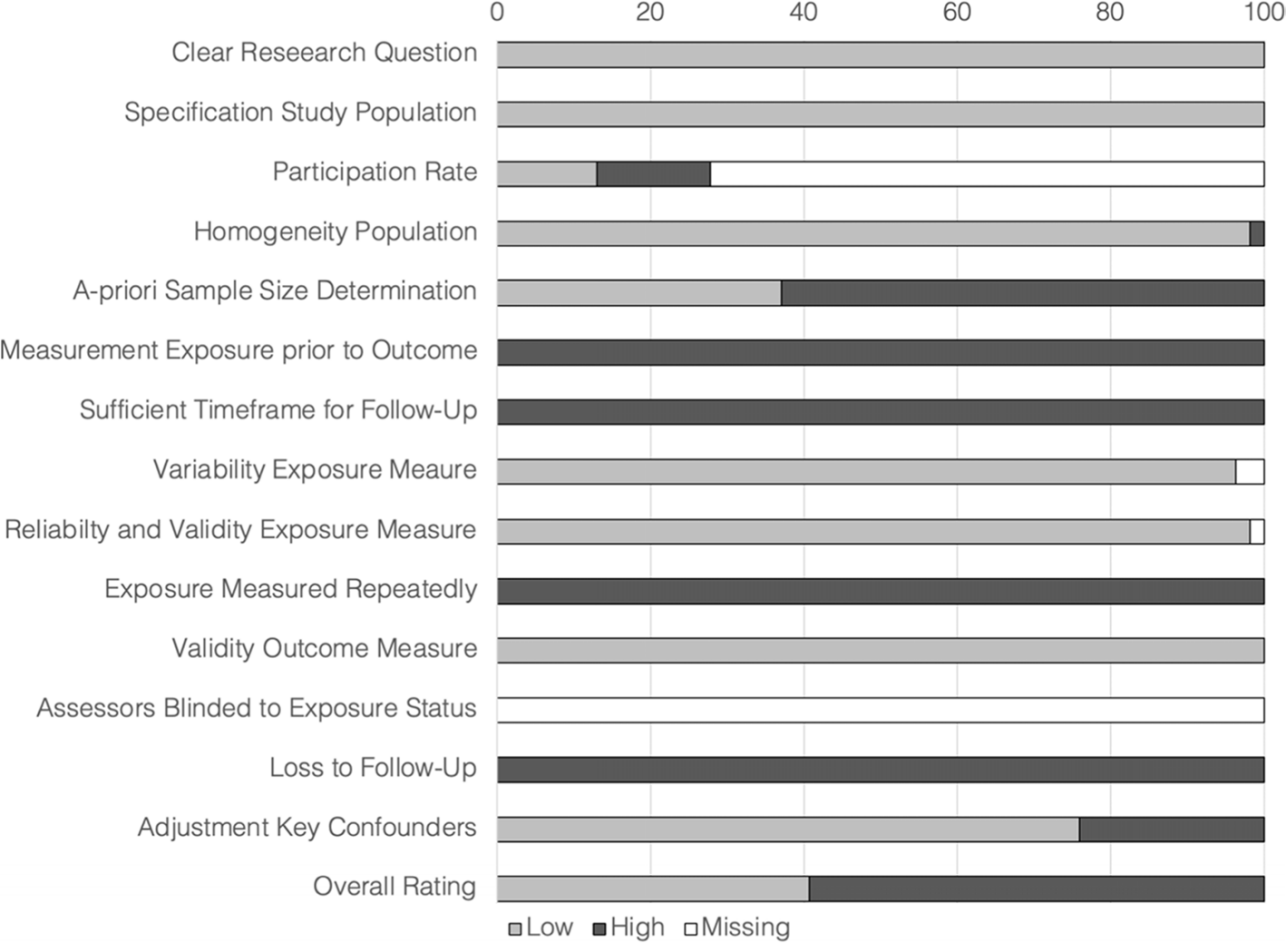
Risk of bias analyses (NIH Quality Assessment Tool)

All included studies were peer-reviewed and we rated the quality of the articles as good or fair (via NIH Quality Assessment Tool). The quality of 22 articles was rated as good and 32 articles as fair (Fig. 2, for more details see Additional file 5). However, the majority of the included articles had a cross-sectional design, presenting a limitation in itself when associated factors or predictors are studied.

### Modifiable predictors of HL

**Fig. 3:**
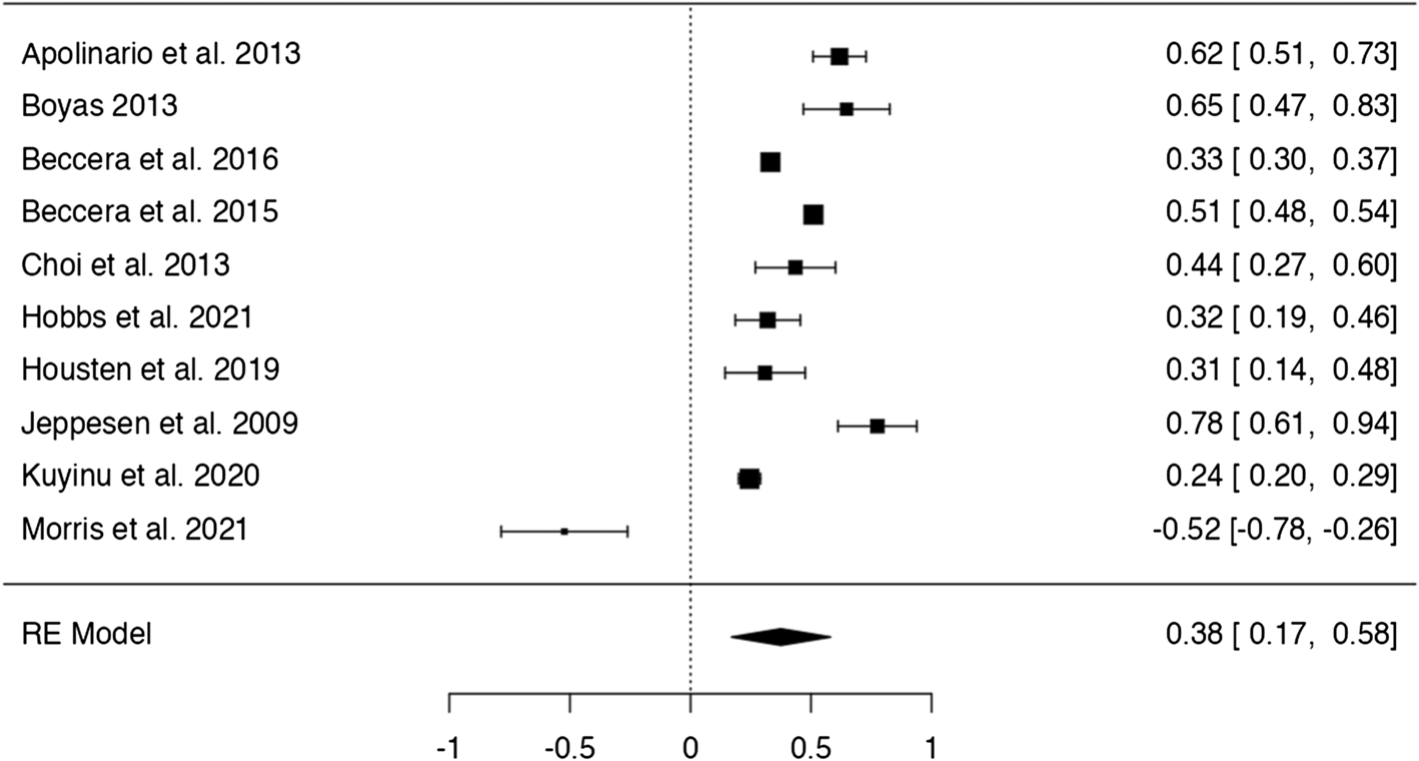
Correlation between language proficiency and health literacy

**Fig. 4:**
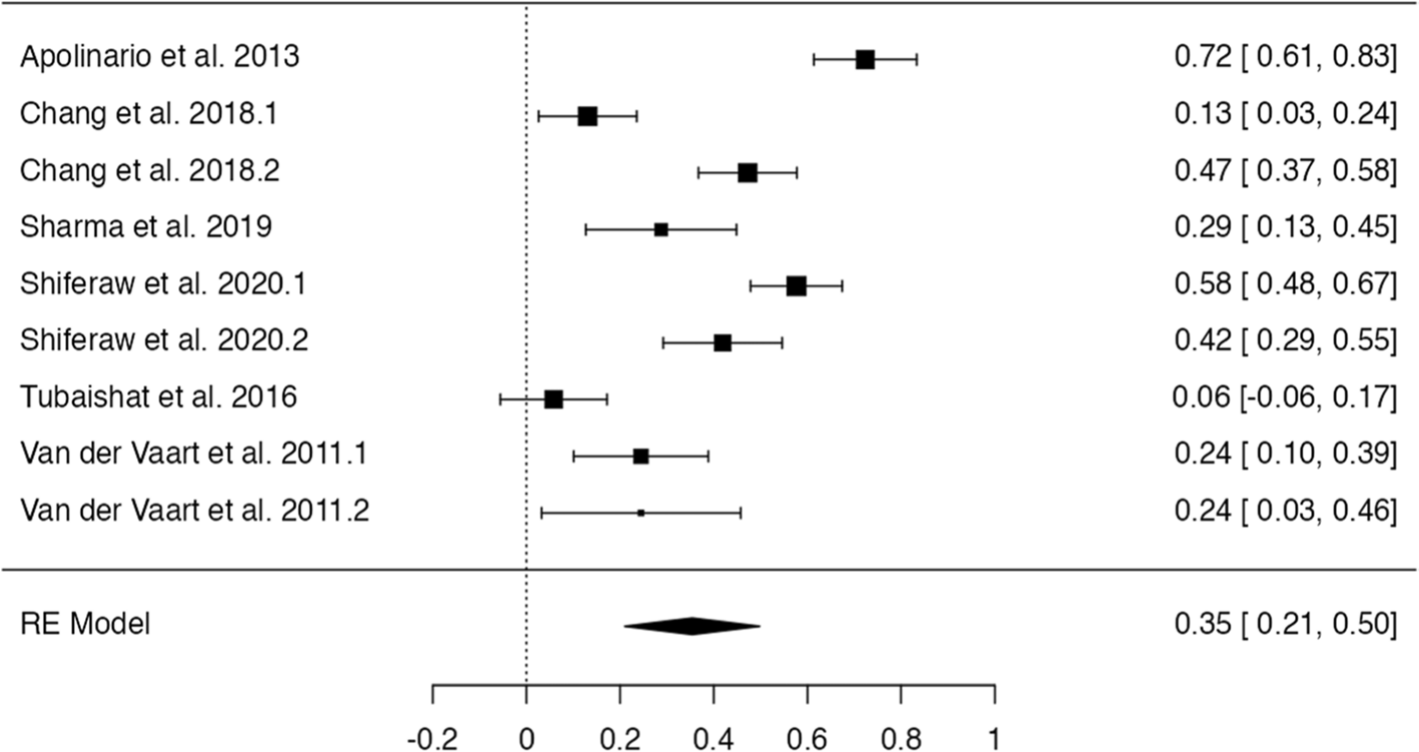
Correlation between frequency of internet use and health literacy

**Fig. 5:**
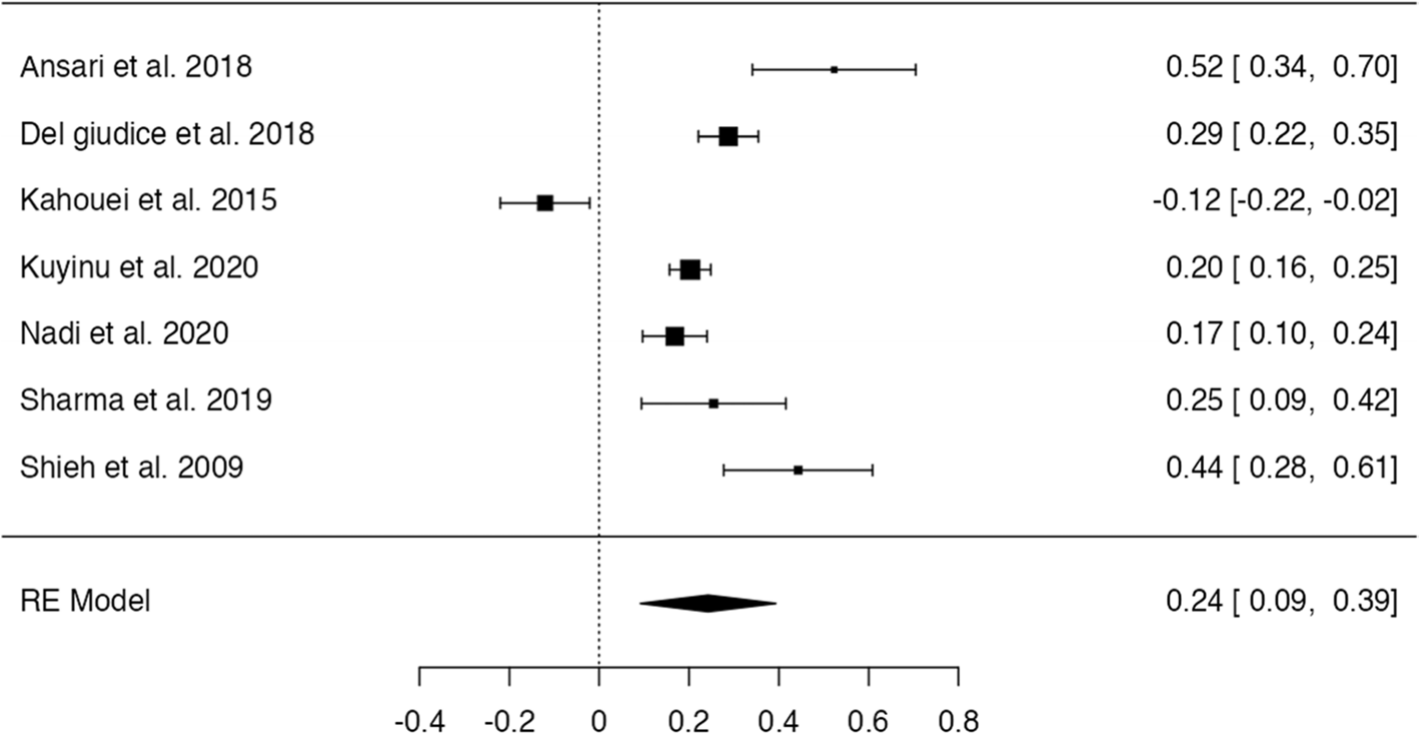
Correlation between using internet as (health) information source and health literacy

**Fig. 6:**
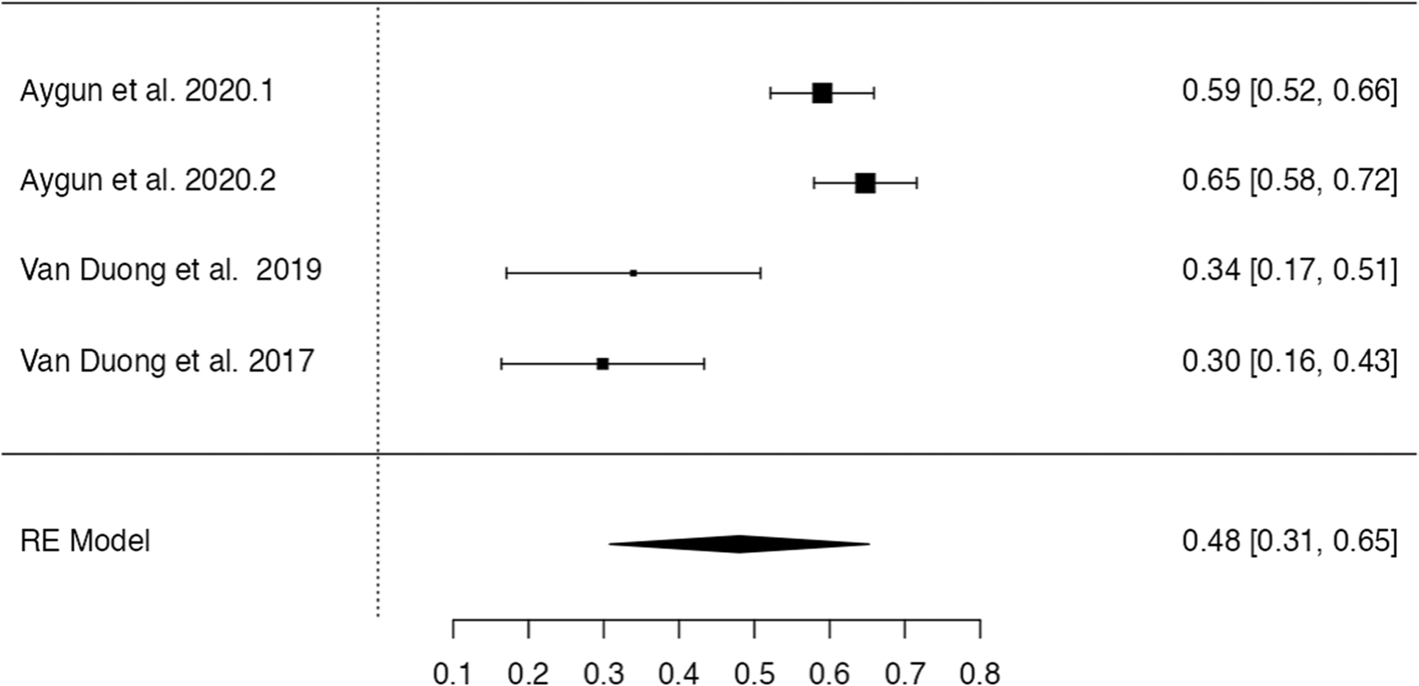
Correlation between consuming health-related TV or reading health news and health literacy

**Fig. 7:**
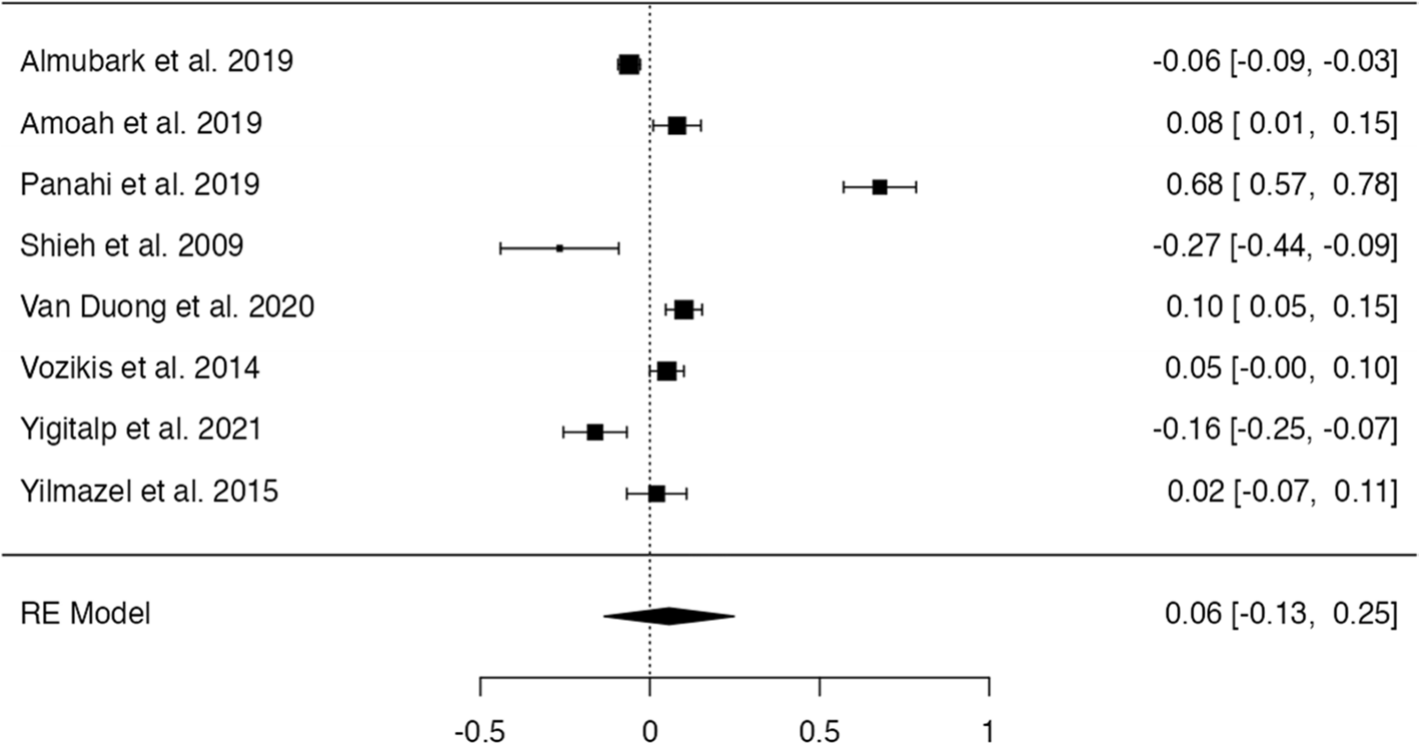
Correlation between not smoking and health literacy

**Fig. 8:**
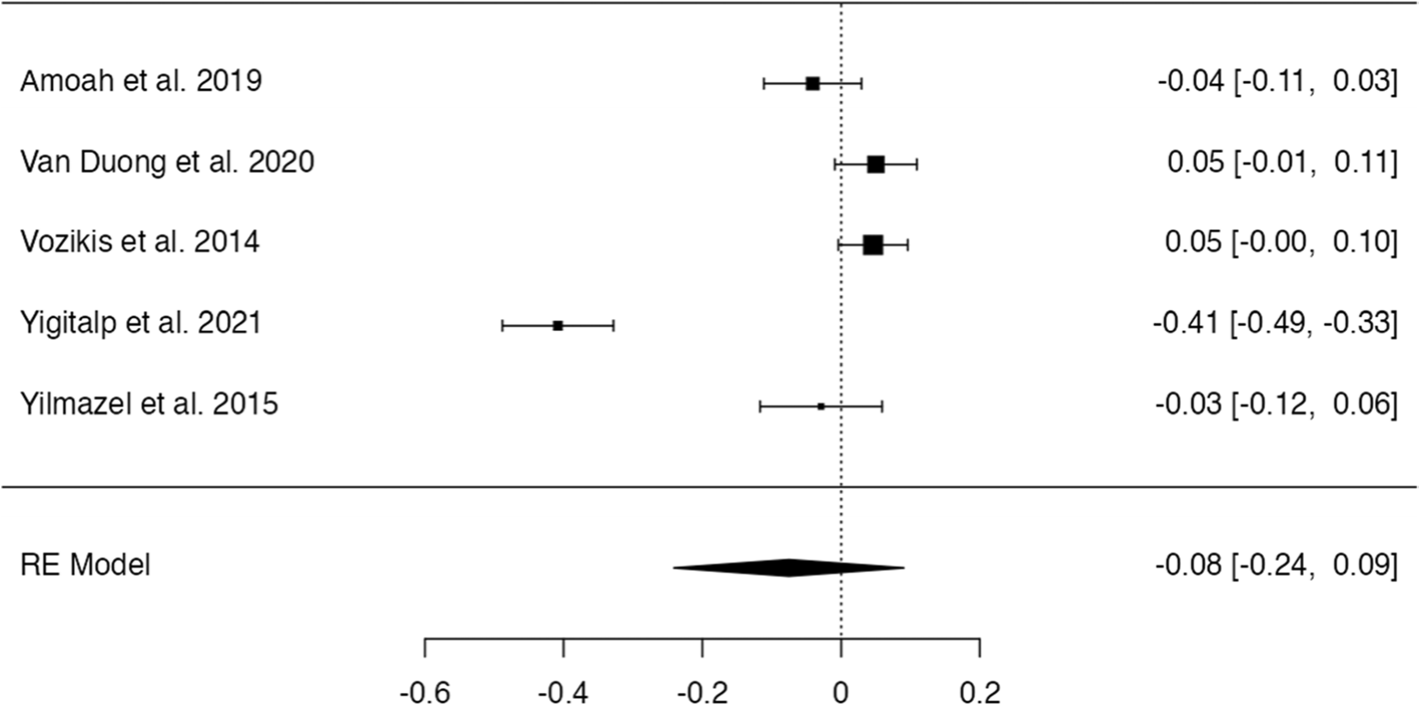
Correlation between low alcohol consumption and health literacy

**Fig. 9:**
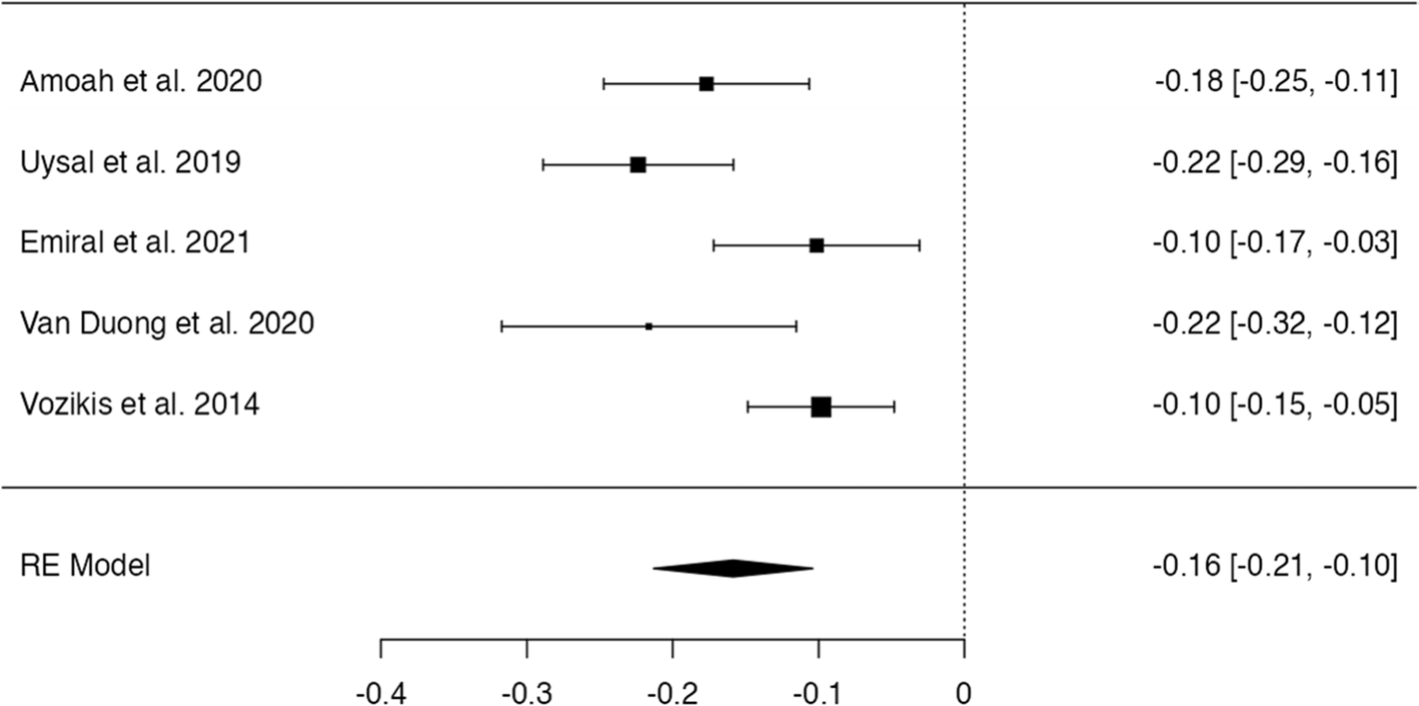
Correlation between not exercising regularly and health literacy

**Fig. 10:**
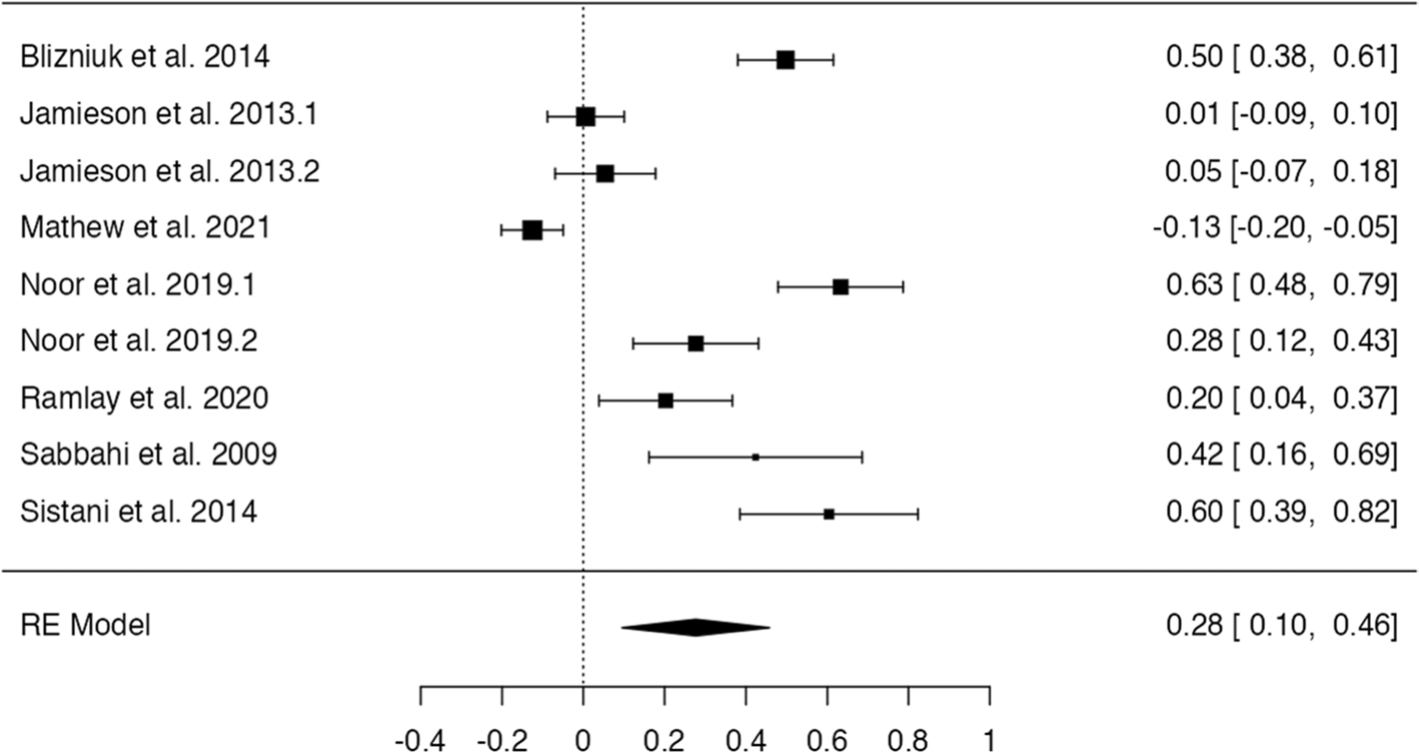
Correlation between regular dental visits/regular brushing behaviour and oral health literacy

**Fig. 11:**
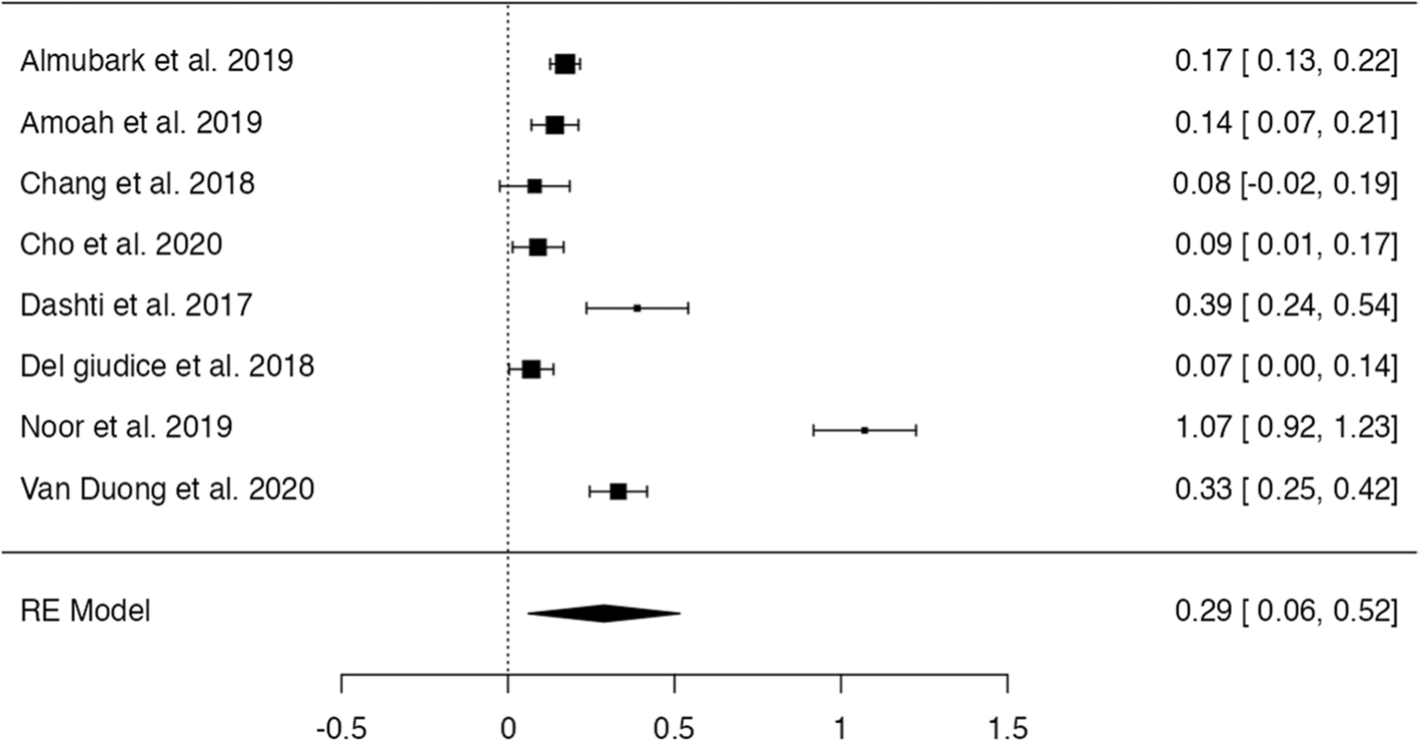
Correlation between good health status and health literacy

In *n* = 54 articles, we found more than 20 potentially modifiable predictors of HL. We included *n* = 41 articles to perform 9 meta-analyses on the associations between modifiable predictors and HL (language proficiency, frequency of internet use, internet as information source, watching health-related TV, smoking, alcohol consumption, physical activity, oral health behaviours and health status) (Figs. 3, 4, 5, 6, 7, 8, 9, 10 and 11). The remaining studies either examined individual predictors exclusively, or the data reported could not be converted to the effect size r. These studies are described narratively (see Additional file 1).

### Language proficiency

Ten studies examined the relationship between language proficiency and HL (Fig. 3), and the z-standardized correlation coefficients ranged from − 0.52 to 0.78 with 9 studies showing a positive association of high language proficiency with adequate HL. The pooled correlation was significant and positive, *r* = 0.38 [95% CI: 0.17, 0.58], and the Q-test suggests significant heterogeneity between studies (Q(9) = 200.69 *p* < 0.0001, tau^2^=0.11, I^2^ = 98.81%). One study (Morris et al. 2021) [28] had a studentized residual larger than ±2.81 and may be a potential outlier and might be overly influential according to Cook’s distances. There was no indication of funnel plot asymmetry (see Additional file 6).

### Frequency of internet use and computer skills

Six studies and nine analyses examined the relationship between frequency of internet use/computer skills and HL (Fig. 4) and the z-standardized correlation coefficients ranged from 0.06 to 0.72. All studies showed a positive association between high frequency of internet use and adequate HL with a pooled correlation coefficient of *r* = 0.35 [95% CI: 0.21, 0.50]. There was significant heterogeneity between studies, (Q(8) = 114.98, *p* < 0.0001, tau^2^=0.04, I^2^ = 91.93%). No outliers or overly influential studies were identified. There was no indication of funnel plot asymmetry (see Additional file 6).

### Using the Internet as a source of health information/ for health-related purposes

Seven studies examined the relationship between using the internet as health information source and HL (Fig. 5) with six studies showing a positive association between using internet as an information source and adequate HL. The z-standardized correlation coefficients ranged from − 0.12 to 0.52 and the pooled correlation coefficient was *r* = 0.24 [95% CI: 0.09, 0.39]. The Q-test indicated significant heterogeneity (Q(6) = 67.84, *p* < 0.0001, tau2=0.04, I2 = 95.07%). One study [34] had a studentized residual larger than ±2.69 and may be a potential outlier, none of the studies was overly influential and no indication of funnel plot asymmetry was given (see Additional file 6).

### Watching health-related TV

Four analyses of three studies examined the relationship between watching health-related TV or reading health news and HL (Fig. 6), all showing a positive association with HL. Z-standardized correlation coefficients ranged from 0.30 to 0.65, and a pooled r of 0.48 [95% CI: 0.31, 0.65] was found. The Q-test showed significant heterogeneity. No outliers or overly influential studies were identified. The regression test (*p* < 0.0001), but not the rank correlation test (*p* = 0.33) indicated funnel plot asymmetry (see Additional file 6).

### Smoking behaviour

A total of eight studies examined the association between not smoking and HL (Fig. 7). Results differed substantially across the studies with z-standardized correlation coefficients ranging from − 0.27 to 0.68, and a non-significant pooled correlation coefficient of *r* = − 0.06 [95% CI: − 0.13, 0.25]. The Q-test suggests significant heterogeneity (Q(7) = 205.38, *p* < 0.0001, tau2=0.07, I2 = 98.57%). One study [36] had a residual larger than ±2.73 and may be a potential outlier. No study was considered to be overly influential. There was no indication for funnel plot asymmetry.

### Alcohol consumption

Five studies examined the relationship between alcohol consumption and HL (Fig. 8), showing no statistically significant association between low alcohol consumption and HL. The z-standardized correlation coefficients ranged from − 0.41 to 0.05 with a pooled r of − 0.08 [95% CI: − 0.24, 0.09]. The Q-test suggests significant heterogeneity (Q(4) = 101.12, *p* < 0.0001, tau2=0.03, I2 = 96.73%). One study [42] may be a potential outlier according to residually, and might be overly influential according to Cook’s distances. There was no indication for funnel plot asymmetry.

### Physical activity

All five studies examining the relationship between not exercising regularly and HL (Fig. 9) indicated a negative correlation, whereby lack of regular exercise was associated with lower HL with z-standardized correlation coefficients ranging from − 0.22 to − 0.10 and a pooled correlation coefficient of *r* = − 0.16 [95% CI: − 0.21, − 0.10]. According to the Q-test, correlations were heterogeneous (Q(4) = 13, *p* = 0.01, tau^2^=0.003, I^2^ = 68.04%). No outliers or overly influential studies were identified and there was no indication for funnel plot asymmetry (see Additional file 6).

### Oral health behaviour (dental visits and tooth-brushing behaviour)

Nine analyses in seven studies examined the relationship between oral health behaviours (brushing frequently or dental visits) and oral health literacy (Fig. 10), showing a positive association between oral health behaviours and oral health literacy with z-standardized correlation coefficients ranging from − 0.13 to 0.63 and a pooled r of 0.28 [95% CI: 0.10, 0.46]. The Q-test indicated significant heterogeneity (Q(8) = 156.26, *p* < 0.0001, tau2=0.07, I2 = 94.18%). There was no indication of outliers or overly influential studies. Funnel plot asymmetry was suggested by the regression test (*p* = 0.02) but not the rank correlation test (p = 0.12) (see Additional file 6).

### Health status

All eight studies on the relationship between health status and HL showed a positive association between ‘good ‘health and adequate HL (Fig. 11). The z-standardized correlation coefficients ranged from 0.07 to 1.07, and the pooled effect was calculated as *r* = 0.29 [95% CI: 0.06, 0.52]. The Q-test indicated significant heterogeneity (Q(7) = 168.19, *p* < 0.0001, tau^2^=0.07, I^2^ = 98.57%). One study (Noor et al. 2019) [69] may be a potential outlier based on residuals and overly influential according to Cook’s distances. The regression test indicated funnel plot asymmetry (*p* = 0.08) but not the rank correlation test (*p* = 0.18) (see Additional file 6).

### Further modifiable predictors: narrative review

Three studies reported multivariate analyses only and thus could not be considered for the meta-analyses. Michou et al. reported a significant association between not smoking, not drinking alcohol, being physical active and adequate HL [66]. Kayupova et al. found an association of HL with low frequency of watching health-related TV in a multivariate linear regression model [61] and Shah et al. identified Body Mass Index (BMI) as being associated with HL using a logistic regression model [29]. BMI was also identified being associated with HL in two further studies [40, 64].

A number of additional modifiable predictors were examined in one study only. All examinations converged in that higher levels of these factors were associated with higher levels of HL. Some of these factors were study time [32], ,last cervical cancer pap test within 36 months [70], being member of a social organization [26], engaging in social groups [27], better information/access to books [60], adherence to Mediterranean diet [47], use of specific medical websites [33] and being a member of health club/welfare group in their community within last 6 months [52] (see Additional file 1 for summary of all study findings).

### Sensitivity analyses

In six articles we could not identify if the study population matched our inclusion criteria. We looked at them separately and found similar results as in our main findings. These were: English proficiency, consuming all types of information on the internet and using more search strategies while looking for the information on the internet. Poor health behaviors as smoking, drinking alcohol, having a higher BMI and physical inactivity were associated with lower HL scores (see Additional file 3 for more details).

## Discussion

This rapid review examined the associations between HL and potentially modifiable predictors in working-age adults. We found significant pooled associations between HL and the following variables: being proficient in a country’s majority language, using the internet more frequently in general, watching more health-related TV, explicitly using internet as an information source or for health-related purposes more often, being more physically active and having a better health status. However, smoking or drinking were not associated with HL. Further, regular dental visits and regular brushing behaviour were associated with oral health literacy.

While the meta-analyses that we conducted summarized bivariate relationships between HL and the factors reported above, several studies also examined these predictors in multivariate analyses and confirmed the direction and strength of these relationships. This means that the relationships between HL and these predictors remained more or less stable, even if controlled for other variables in multivariate models.

### Language proficiency

Consistent with other studies, this review demonstrated that higher proficiency in the majority language of a country was associated with higher HL. In particular if HL is operationalized as the ability to search for, understand and act upon health-related information [2], this association is hardly surprising. However, at the same time this result has important implications with regard to improving HL in different directions.

On the one hand, it would suggest programmes to improve language proficiency in populations with low HL that are comprised of a substantial proportion of non-native language speakers. While this is promising, there are known barriers that limit the potential of such programmes to promote literacy and language skills in migrant and refugee populations. The number of language programmes often is insufficient [71], and disadvantage as well as material circumstances resulting from migration or refugee status such as family issues, home, mobility issues (especially refugees often have to change accommodations) and cultural diversity limits the reach and the acceptability of language-only programmes [71]. If language proficiency programmes are available, contextualized phonics teaching and oral skills development should be focused on [72]. Realistic language materials that relate to daily life conversations activities and personal documents could be further effective ways of promoting language skills [73]. Providing access to internet classes, improving internet skills and providing health-related information online can be useful methods to improve HL [74].

On the other hand, the relationship between language proficiency and HL points to the potential of providing easily accessible health-related information in multiple languages, particularly those spoken by ethnic minorities, immigrant and refugee population in the respective community, as well as potentially simplified language. Language barriers are conceptually unrelated to the processing capacities implied in the HL concept. Therefore, they could be easily addressed by providing information in languages that are actually used by the populations for whom health information is being provided.

### Internet usage

Our rapid review found a medium-sized association between frequency of internet usage (both general and for health-related information in particular) and HL, suggesting that easy access to health-related information through the internet could facilitate HL. At the same time, the amount of imprecise, ambiguous and purposely misleading information in the internet suggests that critical appraisal skills will also be necessary that enable users to estimate both the veracity and trustworthiness of health-related content. As some of the studies included in the meta-analyses that examined the relationship between internet use and HL have been conducted in the 2000s, the increase of the sheer amount of information and misinformation in the last years would suggest that digital HL [75] is becoming increasingly important. In fact, a recent study in Germany [76] suggests that digital HL is distributed along the same socioeconomic fault lines as HL, which puts the same populations at risk for inadequate digital HL that are already at risk for low overall HL.

### Health behaviours & health status

We found higher levels of physical activity, better health status and better oral health – related behaviours to be associated with higher levels of HL. Numerous studies have investigated the reverse direction of a potential exposure-effect relationship and found positive associations of HL with a range of health-related behaviours e.g. [77, 78]. Our findings suggest that engaging in health-related behaviours could also motivate higher interest in health-related issues in general, which in turn could have implications for interventions – by increasing activity in terms of health-related behaviours, populations might also increase their HL levels. The underlying processes are unclear, and several theoretical concepts may apply. A pathway in line with autonomous motivation (e.g. [79]) is conceivable: those who experience being able to engage in health-related behaviours might also increase their interest and motivation towards health in general and thus experience increases in HL. This would suggest that interventions to support health behaviours could also impact on HL levels.

### Other determinants

We found a range of other potentially modifiable predictors of HL including adherence to Mediterranean diet, engaging in social groups, being member of a social organization etc. However, the scarcity of studies precluded any approach to evidence synthesis, and we refrain from interpreting these associations.

### Implications for research and practice

The main factors identified here – language and use of digital information – point to the potential of providing health-related information in different languages (especially for immigrant populations), promoting language skills in low-literate populations while offering language courses [71] and improving digital literacy via computer courses. More research is needed to disentangle the interdependent relationships (“chicken-and-egg-conundrum”) between health behaviours and health literacy.

### Strengths and limitations

The majority of the included articles had a very similar study structure including study designs and objectives, which allowed summarizing results in meta-analyses. However, the study populations were heterogeneous (see Additional file 4), and both the health literacy concepts (general vs. domain-specific) and the measurement instruments used in the studies were heterogeneous as well. This means that some populations might have higher levels of health literacy than others, for example, it is conceivable that school teachers [43, 52] might have higher levels of health literacy on average as compared to immigrant populations [20, 21]. However, due to the heterogeneity in assessment instruments and the resulting small cell sizes, we could not formally test such potential differences. Due to our search strategy, the assessed outcomes of the analysed articles were relatively consistent in referring to some type of HL (general HL, oral HL, e-HL or mental HL).

Almost all included studies in this review employed cross-sectional research designs. This makes it impossible to distinguish correlation from causation, and from examining potential feedback loops between HL and health outcomes. In order to interpret such associations as causal, stronger research designs with cohort studies and repeated measurements of HL and potentially modifiable factors are needed.

## Conclusion

This rapid review with meta-analyses identified associations between potentially modifiable downstream factors and HL in working-age populations. This population is particularly relevant, as subgroups within this population, in particular those with long-term unemployment are at particular high risk for low HL.

We found associations between language skills, internet use, internet as information source and good health behaviours and HL. Even though the designs of the studies reviewed do not allow causal interpretations, these variables provide targets for both structural and individual-level interventions to improve HL.

## Supplementary Information

**Additional file 1.** Articles included in the rapid review and data extraction table.

**Additional file 2.** Analysed variables for the meta-analyses.

**Additional file 3.** Sensitivity analyses.

**Additional file 4.** Health literacy of specific populations.

**Additional file 5. **Risk of bias analysis.

**Additional file 6. **Meta-analysis and heterogeneity analysis.

## Data Availability

All data generated or analysed during this study are included in this published article and its supplementary information files.

## Footnotes

1 The WHO first introduced the concept of “health literacy” in 1998

2 For studies not reporting age range, we defined this pragmatically as 65 < = M + 1SD of the sample age distribution
