# Supplementary material for: Modifiable predictors of health literacy in working-age adults - a rapid review and meta-analysis
Source: BMC Public Health. 2022 Jul 30;22:1450. doi: 10.1186/s12889-022-13851-0 (PMC9338662; doi:10.1186/s12889-022-13851-0)
Supplement: Supplementary file 1 — Additional file 1. Articles included in the rapid review and data extraction table. [file 12889_2022_13851_MOESM1_ESM.docx]

# **Additional file 1: Articles included in the rapid review and data extraction table**

Modifiable predictors of health literacy in working-age adults - A rapid review and meta-analysis

Almubark, R., M. Basyouni, A. Alghanem, N. Althumairi, D. Alkhamis, L. S. Alharbi, N. Alammari, A. Algabbani, F. Alnofal, A. Alqahtani and N. BinDhim (2019). "Health literacy in Saudi Arabia: Implications for public health and healthcare access." Pharmacol Res Perspect **7**(4): e00514.

Amoah, P. A. and D. R. Phillips (2020). "Socio-demographic and behavioral correlates of health literacy: a gender perspective in Ghana." Women Health **60**(2): 123-139.

Ansari, M., F. Mohammad-moradi, M. Khaledian, M. Shekofteh, A. Karimi and A. Valinejadi (2018). "Assessment of health literacy level in 18-30 year-old adults, An Iranian experience." Library Philosophy and Practice **2018:** 7.

Apolinario, D., L. L. Mansur, M. T. Carthery-Goulart, S. M. Brucki and R. Nitrini (2013). "Detecting limited health literacy in Brazil: development of a multidimensional screening tool." Health Promot Int **29**(1): 5-14.

Aygun, O. and S. Cerim (2020). "The relationship between general health behaviors and general health literacy levels in the Turkish population." Health Promot Int. 2020 **36**(5): 1275-1289.

Bazaz, M., P. Shahry, S. M. Latifi and M. Araban (2019). "Cervical Cancer Literacy in Women of Reproductive Age and Its Related Factors." J Cancer Educ **34**(1): 82-89.

Becerra, B. J., D. Arias and M. B. Becerra (2016). "Low Health Literacy among Immigrant Hispanics." J Racial Ethn Health Disparities **4**(3): 480-483.

Becerra, M. B., B. J. Becerra, G. P. Daus and L. R. Martin (2015). "Determinants of Low Health Literacy Among Asian-American and Pacific Islanders in California." J Racial Ethn Health Disparities **2**(2): 267-273.

Blizniuk, A., M. Ueno, S. Furukawa and Y. Kawaguchi (2014). "Evaluation of a Russian version of the oral health literacy instrument (OHLI)." BMC Oral Health **14**: 141.

Boyas, J. F. (2013). "Correlates of health literacy among latinos in Arkansas." Social Work in Public Health **28**(1): 32-43.

Chang, A. and P. J. Schulz (2018). "The Measurements and an Elaborated Understanding of Chinese eHealth Literacy (C-eHEALS) in Chronic Patients in China." Int J Environ Res Public Health **15**(7): 1553.

Cho, M., Y.-M. Lee, S. j. Lim and H. Lee (2020). "Factors Associated with the Health Literacy on Social Determinants of Health: A Focus on Socioeconomic Position and Work Environment." International Journal of Environmental Research and Public Health **17**(18): 6663.

Choi, S. E., E. Rush and S. Henry (2013). "Health literacy in Korean immigrants at risk for type 2 diabetes." J Immigr Minor Health **15**(3): 553-559.

Dashti, S., N. Peyman, M. Tajfard and H. Esmaeeli (2017). "E-Health literacy of medical and health sciences university students in Mashhad, Iran in 2016: a pilot study." Electron Physician **9**(3): 3966-3973.

Del Giudice, P., G. Bravo, M. Poletto, A. De Odorico, A. Conte, L. Brunelli, L. Arnoldo and S. Brusaferro (2018). "Correlation Between eHealth Literacy and Health Literacy Using the eHealth Literacy Scale and Real-Life Experiences in the Health Sector as a Proxy Measure of Functional Health Literacy: Cross-Sectional Web-Based Survey." J Med Internet Res **20**(10): e281.

Denuwara, H. and N. S. Gunawardena (2017). "Level of health literacy and factors associated with it among school teachers in an education zone in Colombo, Sri Lanka." BMC Public Health **17**(1): 631.

Emiral, G. O., M. Tozun, B. I. Atalay, S. Goktas, G. Dagtekin, H. Aygar, D. Arslantas, A. Unsal, A. B. Babaoglu and K. Tirpan (2021). "Assessment of knowledge of metabolic syndrome and health literacy level among adults in Western Turkey." Niger J Clin Pract **24**(1): 28-37.

Gallè, F., P. Calella, C. Napoli, F. Liguori, E. A. Parisi, G. B. Orsi, G. Liguori and G. Valerio (2020). "Are Health Literacy and Lifestyle of Undergraduates Related to the Educational Field? An Italian Survey." Int J Environ Res Public Health **17**(18): 6654.

Gantz, L., A. Calvo, M. Hess-Holtz, F. Gonzales, L. Alguero, S. Murphy, M. Moran, L. B. Frank, J. S. Chatterjee, P. Amezola de Herrera and L. Baezconde-Garbanati (2019). "Predictors of HPV Knowledge and HPV Vaccine Awareness Among Women in Panama City, Panama." World Medical and Health Policy **11**(1): 95-118.

Hobbs, K., D. M. Muscat, D. Ceprnja, J. A. Gibson, C. Blumenthal, R. Milad, C. Burns, S. Dennis, T. Lau and V. Flood (2021). "Assessing health literacy among adult outpatients attending allied health clinics in western sydney: A cross-sectional survey using a multidimensional instrument." Health Promot J Austr. **33**(1): 83-90.

Housten, A. J., D. S. Hoover, V. Correa-Fernández, L. L. Strong, W. L. Heppner, C. Vinci, D. W. Wetter, C. A. Spears and Y. Castro (2019). "Associations of Acculturation with English- and Spanish-Language Health Literacy Among Bilingual Latino Adults." Health Lit Res Pract **3**(2): e81-e89.

Jamieson, L. M., K. Divaris, E. J. Parker and J. Y. Lee (2013). "Oral health literacy comparisons between Indigenous Australians and American Indians." Community dental health **30**(1): 52-57.

Jeong, S. H. and H. K. Kim (2016). "Health literacy and barriers to health information seeking: A nationwide survey in South Korea." Patient Educ Couns **99**(11): 1880-1887.

Jeppesen, K. M., J. D. Coyle and W. F. Miser (2009). "Screening questions to predict limited health literacy: a cross-sectional study of patients with diabetes mellitus." Ann Fam Med **7**(1): 24-31.

Kahouei, M., P. S. Roghani, J. M. Zadeh and M. Firouzeh (2015). "The determinants of nursing, allied health and non medical staffs health literacy in hospitals of a developing country." Materia socio-medica **27**(6): 421-424.

Kalkbrenner, M. T., R. E. Flinn, D. K. Sullivan and L. E. Esquivel Arteaga (2021). "A Mental Health Literacy Approach to Supporting First-Generation Community College Student Mental Health: The REDFLAGS Model." Community College Review **49**(3): 243-261.

Kayupova, G., B. Turdaliyeva, K. Tulebayev, T. Van Duong, P. W. Chang and D. Zagulova (2017). "Health Literacy among Visitors of District Polyclinics in Almaty, Kazakhstan." Iran J Public Health **46**(8): 1062-1070.

Kuyinu, Y. A., T. T. Femi-Adebayo, B. I. Adebayo, I. Abdurraheem-Salami and O. O. Odusanya (2020). "Health literacy: Prevalence and determinants in Lagos State, Nigeria." PLoS One **15**(8): e0237813.

Lee, H., J. Hwang, J. Ball, J. Lee and D. Albright (2019). "Is health literacy associated with mental health literacy? Findings from Mental Health Literacy Scale." Perspectives in Psychiatric Care **56**: 393-400.

Mathew, M. A. and Z. Kabir (2021). "Oral health literacy among third-level university students in cork city; Ireland." Ir J Med Sci. **191** (1): 461-467.

Mehay, A. and R. Meek (2021). "Understanding and supporting the health literacy of young men in prison: a mixed-methods study." Health Education **121** (1): 93-110.

Michou, M., D. Panagiotakos, C. Lionis, V. Costarelli and M. Kaur (2020). "Health and Nutrition Literacy in adults: links with lifestyle factors and obesity." Mediterranean Journal of Nutrition and Metabolism **13**: 361-370.

Milner, A., M. Shields and T. King (2019). "The Influence of Masculine Norms and Mental Health on Health Literacy Among Men: Evidence From the Ten to Men Study." Am J Mens Health **13**(5): 1557988319873532.

Morris, N. S., C. Nnaji and M. Sarkis (2021). "Was Test Designed for Africans? Health Literacy and African Immigrants." J Racial Ethn Health Disparities **9**: 315-324.

Noor, N., H. Rani, A. Zakaria, N. Yahya and N. Sockalingam (2019). "Sociodemography, Oral Health Status and Behaviours Related to Oral Health Literacy." Pesquisa Brasileira em Odontopediatria e Clínica Integrada **19**: 1-10.

Nadi, T., J. Poorolajal and A. Doosti-Irani (2020). "Socioeconomic status and health literacy as the important predictors of general health in Iran: A structural equation modeling approach." Epidemiology Biostatistics and Public Health **17(**2): e13312.

Panahi, R., F. Osmani, M. Sahraei, A. Ramezankhani, M. Rezaei, N. Aghaeian, M. Pishvaei, E. Javanmardi and S. Niknami (2019). "The Predictors of Health Literacy Based on the Constructs of Health Belief Model for Smoking Prevention Among University Students." Mod Care J **16**(2): e87068.

Ramlay, M. Z., N. Saddki, M. M. Tin-Oo and W. N. Arifin (2020). "Cross-Cultural Adaptation and Validation of Oral Health Literacy Instrument (OHLI) for Malaysian Adults." Int J Environ Res Public Health **17**(15): 5407.

Sabbahi, D. A., H. P. Lawrence, H. Limeback and I. Rootman (2009). "Development and evaluation of an oral health literacy instrument for adults." Community Dent Oral Epidemiol **37**(5): 451-462.

Shah, L. C., P. West, K. Bremmeyr and R. T. Savoy-Moore (2010). "Health Literacy Instrument in Family Medicine: The “Newest Vital Sign” Ease of Use and Correlates." The Journal of the American Board of Family Medicine **23**(2): 195-203.

Sharma, S., N. Oli and B. Thapa (2019). "Electronic health-literacy skills among nursing students." Adv Med Educ Pract **10**: 527-532.

Shieh, C., R. Mays, A. McDaniel and J. Yu (2009). "Health literacy and its association with the use of information sources and with barriers to information seeking in clinic-based pregnant women." Health Care Women Int **30**(11): 971-988.

Shiferaw, K. B., B. C. Tilahun, B. F. Endehabtu, M. K. Gullslett and S. A. Mengiste (2020). "E-health literacy and associated factors among chronic patients in a low-income country: a cross-sectional survey." BMC Med Inform Decis Mak **20**(1): 181.

Sistani, M. M., A. Montazeri, R. Yazdani and H. Murtomaa (2014). "New oral health literacy instrument for public health: development and pilot testing." J Investig Clin Dent **5**(4): 313-321.

Sistani, M. M., R. Yazdani, J. Virtanen, A. Pakdaman and H. Murtomaa (2013). "Oral health literacy and information sources among adults in Tehran, Iran." Community Dent Health **30**(3): 178-182.

Tubaishat, A. and L. Habiballah (2016). "eHealth literacy among undergraduate nursing students." Nurse Educ Today **42**: 47-52.

Uysal, N., E. Ceylan and A. Koç (2020). "Health literacy level and influencing factors in university students." Health Soc Care Community **28**(2): 505-511.

van der Vaart, R., A. J. van Deursen, C. H. Drossaert, E. Taal, J. A. van Dijk and M. A. van de Laar (2011). "Does the eHealth Literacy Scale (eHEALS) measure what it intends to measure? Validation of a Dutch version of the eHEALS in two adult populations." J Med Internet Res **13**(4): e86.

Van Duong, T., C. H. Chiu, C. Y. Lin, Y. C. Chen, T. C. Wong, P. W. S. Chang and S. H. Yang (2020). "E-healthy diet literacy scale and its relationship with behaviors and health outcomes in Taiwan." Health Promot Int **36**(1): 20-33.

Van Duong, T. V., P. W. Chang, S. H. Yang, M. C. Chen, W. T. Chao, T. Chen, P. Chiao and H. L. Huang (2017). "A New Comprehensive Short-form Health Literacy Survey Tool for Patients in General." Asian Nurs Res (Korean Soc Nurs Sci) **11**(1): 30-35.

Van Duong, T. V., T. T. P. Nguyen, K. M. Pham, K. T. Nguyen, M. H. Giap, T. D. X. Tran, C. X. Nguyen, S. H. Yang and C. T. Su (2019). "Validation of the Short-Form Health Literacy Questionnaire (HLS-SF12) and its determinants among people living in rural areas in Vietnam." Int J Environ Res Public Health **16**(18): 3346.

Vozikis, A., K. Drivas and K. Milioris (2014). "Health literacy among university students in Greece: determinants and association with self-perceived health, health behaviours and health risks." Archives of public health = Archives belges de sante publique **72**(1): 15.

Yiğitalp, G., V. Bayram Değer and S. Çifçi (2021). "Health literacy, health perception and related factors among different ethnic groups: a cross-sectional study in southeastern Turkey." BMC Public Health **21**(1): 1109.

Yılmazel, G. and F. Cetinkaya (2015). "Health literacy among schoolteachers in Çorum, Turkey." Eastern Mediterranean Health Journal **21** (8): 598-605.

| Author and year of publication | Country | Sample size | Type of HL | HL measurement | Modifiable determinants of HL | Results |
| --- | --- | --- | --- | --- | --- | --- |
| Almubark et al. 2019 | Saudi Arabia | N=3557 | General HL | Single‐Item Literacy Screener (Arabic) | Smoking and health status | UVA: Smoking associated with lower HL, better health status associated with higher HL |
| Amoah et al. 2019 | Ghana | N=779 | General HL | European Health Literacy Questionnaire (HLS-EU-Q16) | Smoking and health status | MVA: Smoking associated with lower HL, UVA: better health status with higher HL |
| Ansari et al. 2018 | Iran | N=170 | General HL | Standard Iranian adult HL questionnaire | Health information sources: asking, friends, doctors, internet | UVA: Using specific sources of health information (asking friends, doctors and internet) associated with higher HL |
| Apolinario et al. 2013 | Brazil | N=322 | General HL | Test of functional HL in adults short form (S-TOFHLA (Brazilian) | Use of personal computer, difficulty reading and writing | UVA: Frequent computer use and better reading and writing skills associated with higher HL |
| Aygun et al. 2020 | Turkey | N=826 | General HL | HLS-EU; HLS-TR-Q47 (Turkish) | Reading health news, reading a publication about health | UVA: Often reading health news and often reading publications about health associated with higher HL |
| Bazaz et al. 2017 | Iran | N=231 | Cervical cancer HL | Individual 27 five-choice questions | Health information sources: web searching, mothers’ counseling, friends’ counseling | UVA: Using specific sources of information (web searching, mother´s and friends counseling) associated with better HL |
| Beccera et al. 2016 | USA | N=3061 | General HL | Individual tool (see reference) | English proficiency | UVA: Better English proficiency associated with higher HL |
| Becerra et al. 2015 | USA | N=4045 | General HL | Individual tool (see reference) | English proficiency | UVA: Better English proficiency associated with higher HL |
| Blizniuk et al. 2014 | Belarus | N=281 | OHL | Oral Health Literacy Instrument (OHLI) (Russian) | Regularity of dental visits | UVA: Visiting a dentist at least once a year associated with better OHL |
| Boyas 2013 | USA | N=123 | General HL | Individual tool (see reference) | Linguistic acculturation | UVA: Better lingustic acculturation associated with higher HL |
| Chang et al. 2018 | China | N=352 | E- health literacy | eHealth Literacy Scale (eHEALS) | Media and computer literacy, computer skills, health status | UVA: Better media and computer literacy associated with higher e-HL |
| Cho et al. 2020 | Korea | N=660 | General HL | Health literacy on social determinants of health | Perceived mental health status | UVA: Better perceived mental health status associated with higher HL |
| Choi et al. 2013 | USA | N=145 | General and dietary HL | Newest Vital Sign (NVS) | English proficiency, lower waist to hip ratio | UVA: Better English proficiency and lower waist to hip ratio associated with higher HL |
| Dashti et al. 2017 | Iran | N=192 | E- health literacy | eHEALS (Persian) | Use of specific medical websites, health status | UVA: Better health status and use of medical websites associated to higher e-HL |
| Del Guidice et al. 2018 | Italy | N=868 | E- health literacy | eHEALS (Italian) | Internet use for health, self-rated health | UVA: Frequent internet use and better self-rated health associated to hiher e-HL |
| Denuwara et al. 2017 | Sri Lanka | N=502 | General HL | HLS-EU-Scale | Being member of health club/welfare groups, participation in health-related course, print media as general and health information source | UVA: Not being a member of a health club, no participation in a health-related courses, and not using print media as general and health information source associated with lower HL |
| Emiral et al. 2021 | Turkey | N=774 | General HL | HLS-EU-Q16 | Physical activity, viewing television less than 3 hours a day, not having optimal BMI | UVA: Higher physical activity, viewing television less than 3 hours a day and not being obese associated with higher HL |
| Galle et al. 2020 | Italy | N=806 | General HL | Health Literacy Assessment Tool and NVS | Adherence to Mediterranean diet (MD) | UVA: Better adherence to Mediterrenean diet associated with higher HL |
| Gantz et al. 2019 | Panama | N=333 | HPV knowledge | Individual tool (see reference) | Last Pap test within past 36 months | UVA: Having completed last pap test within past 36 months associated with higher HL |
| Hobbs et al. 2021 | Australia | N=230 | General HL | Health Literacy Questionnaire | Not speaking English at home | UVA: Not speaking English at home associated with lower HL |
| Housten et al. 2019 | USA | N=142 | General HL | REALM and SAHLSA | English and Spanish profciency | UVA: Better English and Spanish proficiency associated with higher HL |
| Jamieson et al. 2013 | Australia and USA | N=468 (study 1) N=254 (study 2) | OHL | REALD-30 instrument | Infrequent dental attendance | UVA: Infrequent dental attendance associated with lower OHL |
| Jeong et al. 2016 | South Korea | N=1000 | General HL | NVS (Korean) | Barrier regarding how to get information and regarding access to expensive books and magazines | UVA: Barriers regarding how to get information and barriers regarding access to expensive books and magazines associated with lower HL |
| Jeppesen et al. 2009 | USA | N=225 | General HL | S-TOFHLA | Poor reading and writing skills | UVA: Poor self-rated reading ability associated to lower HL |
| Kahouei et al. 2015 | Iran | N=389 | General HL | Iranian HL measurement | Use information sources: TV, internet, physicians and books and journals | UVA:Using specific information sources (TV, internet, physicians and books and journals) associated with higher HL |
| Kalkbrenner et al. 2021 | USA | N=294 | MHL | REDFLAGS questionnaire | Being member of a Greek organization | UVA: Being member of a Greek Organization associated with higher MHL |
| Kayupova et al. 2017 | Kazakhstan | N=998 | General HL | HLS-EU-Q47 | Frequency of watching health related TV programs, community involvement | MVA: Low frequency (compared to never) of watching health-related TV and moderate community involvement associated with higher HL |
| Kuyinu et al. 2020 | Nigeria | N=1831 | General HL | Brief Health Literacy Screening tool | Use of the broadcast media or internet information source, English proficiency | UVA: Using specific sources of information (broadcast media, internet) and better English proficiency associated with higher HL |
| Lee et al. 2019 | USA | N=708 | MHL | 13 items of the Mental Health Literacy Scale | Engaging in social groups | UVA: Engaging in social groups asssociated to MHL |
| Mathew et al. 2021 | Ireland | N=663 | OHL | Individual tool (see reference) | Regular dental visits | UVA: Regular dental visits associated with lower OHL |
| Mehay et al. 2021 | Great Britain | N=104 | General HL | HLS-EU-Q47 | Anxiety (HAD-A), depression and lower feelings of positive effect | MVA: Increased anxiety, being depressive and lower feeeling of positive affect associated with lowe HL |
| Michou et al. 2020 | Greece | N=1281 | General HL | HLS-EU-Q47 | Smoking, alcohol consumption, and physical activity | MVA: Smoking (vs ex smokers), not consuming alcohol and high physical activity associated with higher HL |
| Milner et al. 2019 | Australia | N= 8362 | General HL | Health Literacy Questionnaire | Depressive symptoms | UVA: Moderate to severe depressive symptoms associated with lower HL |
| Morris et al. 2021 | USA | N=75 | General HL | NVS, Health Literacy Skills Instrument - Short Form | English proficiency | UVA: Better English proficiency associated with lower HL (via HLSI), not significant with NVS |
| Nadi et al. 2020 | Iran | N=750 | General HL | Individual tool (see reference) | Source of health information: Internet, book or pamphlets, radio & TV, friends and acquaintances | UVA: Use of specific health information sources (internet, book or pamphlets) associated with higher HL and use of radio & TV, friends and acquaintances associated with lower HL |
| Noor et al. 2019 | Malaysia | N=165 | OHL | Health Literacy in Dental Scale (HeLD-14) | Oral health behaviors: oral health status, brushing habit, dental visits | UVA: Good oral health status, brushing teeth regularly and visiting dentist regularly associater with higher OHL |
| Panahi et al. 2019 | Iran | N=337 | General HL | Health Literacy for Iranian Adults Scale | Smoking status | UVA: Smoking associated to lower HL |
| Ramlay et al. 2020 | Malaysia | N=195 | OHL | OHLI (Malay) | Last dental visit | UVA: Last dental visit more than 2 years ago associated to lower HL |
| Sabbahi et al. 2009 | Canada | N=100 | OHL | OHLI | Visiting a dentist | UVA: Regular dental visits associated with higher OHL |
| Shah et al. 2010 | USA | N=808 | General HL | NVS | BMI | MVA: Higher BMI associater with lower HL |
| Sharma et al. 2019 | Nepal | N=152 | E- health literacy | eHEALS | Internet skills and internet for health purposes | UVA: Internet skills and frequency of using internet for health-related purposes associated to e-HL |
| Shieh et al. 2009 | USA | N=143 | General HL | S-TOFHLA | Less likely to use the internet | UVA: Less likely using the internet associated with lower HL |
| Shiferaw et al. 2020 | Ethiopia | N=423 | E- health literacy | eHEALS | Internet use, good knowledge of online resources, computer literacy | UVA: Daily internet use, good knowledge of online resources and better computer literacy associated with higher e-HL |
| Sistani et al. 2014 | Iran | N=97 | OHL | Oral Health Literacy Adult Questionnaire (OHL-AQ) | Brushing behavior | UVA: Frequent toooth brushing associated with higher OHL |
| Sistani et al. 2013 | Iran | N=1030 | OHL | OHL-AQ | Multiple information sources | MVA: Using multiple information sources associated with higher OHL |
| Tubaishat et al. 2016 | Jordan | N=541 | E-health literacy | eHEALS | Internet skills | UVA: Good internet skills associated with higher e-HL |
| Uysal et al. 2019 | Turkey | N=905 | General HL | European Health Literacy Scale | Physical activity, healthy diet | UVA: Higher physical activity and healthy diet associated with higher HL |
| Van der Vaart et al. 2011 | Netherlands | N=189 (study 1) N=88 (study 2) | E- health literacy | eHEALS | Internet use | UVA: Frequent internet use associated with higher e-HL |
| Van Duong et al. 2019 | Vietnam | N=440 | General HL | Short Form Health Literacy Questionnaire (HLS-SF-12) | Watching/hearing health related TV/radio | UVA: Often watching health-related TV associated with higher HL |
| Van Duong et al. 2017 | Taiwan | N=403 | General HL | HLS-SF-12 | Watching health-related TV | UVA: Often watching health-related TV associated with higher HL |
| Van Duong et al. 2020 | Taiwan | N=1342 | General HL and e-healthy diet literacy (e-HDL) | HLS-SF-12, e-HDL | Facebook as information source, health status, smoking status, exercise last 30 days | UVA: Use of specific information source (Facebook), not smoking, better health status and higher physical activity associated with higher HL |
| Vozikis et al. 2014 | Greece | N=1526 | General HL | Individual tool (see reference) | Smoking, alcohol consumption, physical workout | UVA: Smoking and drinking alcohol associated with lower HL, higher physical activity associated with higher HL |
| Yigitalp et al. 2021 | Turkey | N=600 | E- health literacy | European Health Literacy Scale (Turkish) and Health Perception Scale | Health information sources: healthcare professionals, smoking and drinking alcohol | UVA: Use of specific health information sources (healthcare professionals, TV, printed press or internet), smoking and drinking alcohol associated with higher e-HL |
| Yilmazel et al. 2015 | Turkey | N=500 | General HL | NVS | Smoking habit, alcohol use | UVA: Smoking and consuming alcohol associated with lower HL |

HL: health literacy; OHL: Oral health literacy; MHL: Mental health literacy; UNA: Univariate analysis; MVA: Multivariate analysis

*Note*: Primarily univariate analyses reported, MVA reported if univariate analyses were not given, Milner et al. 2019 with a cohort study design, all others with a cross-sectional study design
