# Supplementary material for: Modifiable predictors of health literacy in working-age adults - a rapid review and meta-analysis
Source: BMC Public Health. 2022 Jul 30;22:1450. doi: 10.1186/s12889-022-13851-0 (PMC9338662; doi:10.1186/s12889-022-13851-0)
Supplement: Supplementary file 2 — Additional file 2. Analysed variables for the meta-analyses. [file 12889_2022_13851_MOESM2_ESM.docx]

# **Additional file 2 – Analysed variables for the meta-analyses**

Modifiable predictors of health literacy in working-age adults - A rapid review and meta-analysis

Table 1: Language proficiency

| Author and year of publication | Country of study | Variable | N correlation | Correlation coefficient *r* |
| --- | --- | --- | --- | --- |
| Apolinario et al. 2013 | Brazil | Ability to watch foreign movies with subtitles | 322 | 0,55 |
| Beccera et al. 2015 | USA | English language proficiency | 4045 | 0,47 |
| Beccera et al. 2016 | USA | English language proficiency | 3061 | 0,32 |
| Boyas 2013 | USA | Lingustic acculturation | 123 | 0.57 |
| Choi et al. 2013 | USA | English proficiency | 142 | 0,41 |
| Hobbs et al. 2021 | Australia | Speaking English at home | 213 | 0,31 |
| Housten et al. 2019 | USA | English proficiency | 142 | 0,3 |
| Jeppesen et al. 2009 | USA | Self-rated reading ability | 147 | 0,65 |
| Kuyinu et al. 2020 | Nigeria | Ability to read English | 1842 | 0,24 |
| Morris et al. 2021 | USA | Primary language English | 59 | -0,48 |

Table 2: Frequency of internet use/computer literacy

| Author and year of publication | Country  of study | Variable | N correlation | Correlation coefficient *r* |
| --- | --- | --- | --- | --- |
| Apolinario et al. 2013 | Brazil | Frequency of computer use | 322 | 0,62 |
| Chang et al. 2018 | China | Media & computer literacy | 352 | 0,13 |
| Chang et al. 2018 | China | Computer skills | 352 | 0,44 |
| Sharma et al. 2019 | Nepal | Perceived level of internet skills | 152 | 0,28 |
| Shiferaw et al. 2020 | Ethiopia | Computer literacy | 404 | 0,52 |
| Shiferaw et al. 2020 | Ethiopia | Frequency of internet use | 241 | 0,42 |
| Tubaishat et al. 2016 | Jordan | Frequency of using internet | 299 | 0,06 |
| Van der Vaart et al. 2011 | Netherlands | Amount of internet usage (study 1 patients with rhematic diseases) | 189 | 0,24 |
| Van der Vaart et al. 2011 | Netherlands | Amount of internet usage (study 2 sample from regions of Twente) | 88 | 0,24 |

Table 3: Internet as information source

| Author and year of publication | Country of study | Variable | N correlation | Correlation coefficient *r* |
| --- | --- | --- | --- | --- |
| Ansari et al. 2018 | Iran | Health information sources | 119 | 0,48 |
| Del giudice et al. 2018 | Italy | Frequency of internet use for health | 868 | 0,28 |
| Kahouei et al. 2015 | Iran | Internet as information source | 389 | -0,12 |
| Kuyinu et al. 2020 | Nigeria | Frequent sources of information | 1831 | 0,2 |
| Nadi et al. 2020 | Iran | Internet as source of health information | 750 | 0,17 |
| Sharma et al. 2019 | Nepal | Frequency of using internet for health-related purposes | 152 | 0,25 |
| Shieh et al. 2009 | USA | Internet as information source | 143 | 0,42 |

Table 4: Watching health-related TV

| Author and year of publication | Country  of study | Variable | N correlation | Correlation coefficient *r* |
| --- | --- | --- | --- | --- |
| Aygun et al. 2020 | Turkey | Reading health news | 816 | 0,53 |
| Aygun et al. 2020 | Turkey | Reading a publication about health | 826 | 0,57 |
| Van Duong et al. 2019 | Vietnam | Tendency to watch heath related TV/radio more often | 138 | 0,33 |
| Van Duong et al. 2017 | Taiwan | More often watching health-related TV programs | 215 | 0,29 |

Table 5: Smoking habit

| Author and year of publication | Country  of study | Variable | N correlation | Correlation coefficient *r* |
| --- | --- | --- | --- | --- |
| Almubark et al. 2019 | Saudi Arabia | Smoking status | 3557 | -0,06 |
| Amoah et al. 2019 | Ghana | Smoking status | 779 | 0,08 |
| Panahi et al. 2019 | Iran | Smoking status | 337 | 0,59 |
| Shieh et al. 2009 | USA | Current smoking | 129 | -0,26 |
| Van Duong et al. 2020 | Taiwan | Smoking status | 1342 | 0,1 |
| Vozikis et al. 2014 | Greece | Smoking status | 1526 | 0,05 |
| Yigitalp et al. 2021 | Turkey | Smoking status | 442 | -0,16 |
| Yilmazel et al. 2015 | Turkey | Smoking status | 500 | 0,02 |

Table 6: Alcohol consumption

| Author and year of publication | Country  of study | Variable | N correlation | Correlation coefficient *r* |
| --- | --- | --- | --- | --- |
| Amoah et al. 2019 | Ghana | Alcohol consumption yes/no (reference) | 779 | -0,04 |
| Van Duong et al. 2020 | Taiwan | Drink alcohol beverage last 30 days, none, once, twice or more | 1096 | 0,05 |
| Vozikis et al. 2014 | Greece | Alcohol consumption yes/no (reference) | 1526 | 0,05 |
| Yigitalp et al. 2021 | Turkey | Drinking alcohol yes vs no | 600 | -0,39 |
| Yilmazel et al. 2015 | Turkey | Alcohol use, current user vs non-user (reference) | 500 | -0,03 |

Table 7: Physical activity

| Author and year of publication | Country  of study | Variable | N correlation | Correlation coefficient *r* |
| --- | --- | --- | --- | --- |
| Amoah et al. 2019 | Ghana | Exercise | 779 | -0,18 |
| Uysal et al. 2019 | Turkey | Physical activity | 905 | -0,22 |
| Emiral et al. 2021 | Turkey | Doing regular physical activity | 774 | -0,1 |
| Van Duong et al. 2020 | Taiwan | Exercise last 20 days | 380 | -0,21 |
| Vozikis et al. 2014 | Greece | Physical workout | 1526 | -0,1 |

Table 8: Oral health beaviors

| Author and year of publication | Country  of study | Variable | N correlation | Correlation coefficient *r* |
| --- | --- | --- | --- | --- |
| Blizniuk et al. 2014 | Belarus | Regularity of dental visits | 281 | 0,46 |
| Jamieson et al. 2013 | Australia: Indigeneous Australians | Last dental visit < 1 year ago vs ≥ 1 year ago | 431 | 0,01 |
| Jamieson et al. 2013 | American Indians | Last dental visit < 1 year ago vs ≥ 1 year ago | 254 | 0,05 |
| Mathew et al. 2021 | Ireland | Regular dental visit | 663 | -0,13 |
| Noor et al. 2019 | Malaysia | Brushing habit <2 times daily vs 2 times or more daily | 165 | 0,56 |
| Noor et al. 2019 | Malaysia | Dental visit less than once per year vs once or more visit per year | 165 | 0,27 |
| Ramlay et al. 2020 | Malaysia | Last dental visit <1 year vs >2 years/never sought care | 146 | 0,2 |
| Sabbahi et al. 2009 | Canada | Visiting a dentist every 3-6 months vs only when feeling pain | 59 | 0,4 |
| Sistani et al. 2014 | Iran | Brushing behavior Adequate (1–2 times/day) vs never rarely | 83 | 0,54 |

Table 9: Health status

| Author and year of publication | Country  of study | Variable | N correlation | Correlation coefficient *r* |
| --- | --- | --- | --- | --- |
| Almubark et al. 2019 | Saudi Arabia | General health status | 1926 | 0,17 |
| Amoah et al. 2019 | Ghana | General health status | 779 | 0,14 |
| Chang et al. 2018 | China | Health status | 352 | 0.08 |
| Cho et al. 2020 | Korea | Perceived mental health status | 660 | 0.09 |
| Dashti et al. 2017 | Iran | Health status | 168 | 0,37 |
| Del giudice et al. 2018 | Italy | Self-rated health | 868 | 0,07 |
| Noor et al. 2019 | Malaysia | Oral health status | 165 | 0,79 |
| Van Duong et al. 2020 | Taiwan | Health status | 518 | 0,32 |
