# Supplementary material for: Modifiable predictors of health literacy in working-age adults - a rapid review and meta-analysis
Source: BMC Public Health. 2022 Jul 30;22:1450. doi: 10.1186/s12889-022-13851-0 (PMC9338662; doi:10.1186/s12889-022-13851-0)
Supplement: Supplementary file 3 — Additional file 3. Sensitivity analyses. [file 12889_2022_13851_MOESM3_ESM.docx]

**Additional file 3 – Sensitivity analyses**

Modifiable predictors of health literacy in working-age adults - A rapid review and meta-analysis

Six articles excluded during the full-text screening did not report the mean age of the population but only age groups or the age range which was not between 18 and 65 years. As we could not clearly identify if the study population matched our inclusion criteria, we looked at these articles separately and compared the predictors reported in them with our main findings.

The health literacy (HL) determinants reported in the studies from Australia (1), USA (2), Serbia (3), Israel (4), Denmark (5) and China (6) were similar to the ones found in the articles included in the main analysis.

Adequate HL and e-health literacy were predicted by English proficiency (2), consuming all types of information on the internet and using more search strategies while looking for information on the internet (4), contraceptive use, knowledge of the human papillomavirus and following health-related topics (3). Poor health behaviors as smoking (1), drinking alcohol (1) and having a higher BMI (1, 6) and physical inactivity (5) were associated with lower HL scores.

# References

1. Hosking SM, Brennan-Olsen SL, Beauchamp A, Buchbinder R, Williams LJ, Pasco JA. Health literacy in a population-based sample of Australian women: a cross-sectional profile of the Geelong Osteoporosis Study. BMC Public Health. 2018;18(1):876.

2. Jacobson HE, Hund L, Soto Mas F. Predictors of English Health Literacy among U.S. Hispanic Immigrants: The importance of language, bilingualism and sociolinguistic environment. Lit Numer Stud. 2016;24(1):43-64.

3. Maricic M, Stojanovic G, Pazun V, Stepović M, Djordjevic O, Macuzic IZ, et al. Relationship Between Socio-Demographic Characteristics, Reproductive Health Behaviors, and Health Literacy of Women in Serbia. Front Public Health. 2021;9:629051.

4. Neter E, Brainin E. eHealth literacy: extending the digital divide to the realm of health information. J Med Internet Res. 2012;14(1):e19.

5. Svendsen MT, Bak CK, Sørensen K, Pelikan J, Riddersholm SJ, Skals RK, et al. Associations of health literacy with socioeconomic position, health risk behavior, and health status: a large national population-based survey among Danish adults. BMC Public Health. 2020;20(1):565.

6. Xie Y, Ma M, Zhang Yn, Tan X. Factors associated with health literacy in rural areas of Central China: structural equation model. BMC Health Services Research. 2019;19(1):300.
