# Supplementary material for: Modifiable predictors of health literacy in working-age adults - a rapid review and meta-analysis
Source: BMC Public Health. 2022 Jul 30;22:1450. doi: 10.1186/s12889-022-13851-0 (PMC9338662; doi:10.1186/s12889-022-13851-0)
Supplement: Supplementary file 4 — Additional file 4. Health literacy of specific populations. [file 12889_2022_13851_MOESM4_ESM.docx]

# **Additional file 4 - Health literacy of specific populations**

We compared health literacy (HL) across specific populations. It needs to be considered that HL measurement tools were heterogeneous and difficult to compare, the results need to be interpreted cautiously. Still, we provided HL means of the specific populations as given in the studies. If mean scores were not given, HL categories represented most often were reported.

In university students, on average adequate HL was shown in one study, moderate HL was shown in three studies, and limited HL in one study. For the remaining studies, no fixed cut-off was given (Table 1) (1-7). Among teachers, one study showed on average sufficient HL (8) and one study showed limited HL (9).

In clinical populations, two studies reported adequate HL in majority of the participants. In both studies, HL was measured through the S-TOFHLA (abbreviated version of Test of Functional Health Literacy in Adults). Furthermore, high OHL means in two studies indicated adequate OHL levels (10, 11). In general adult populations, three studies reported sufficient or adequate HL levels (12-14) and four limited HL (15-18).

Table 1: Health literacy (HL) levels sorted by specific populations

| **Author and year of publication** | **Country** | **Study design** | **Definition of study population** | **Sample size** | **Type of HL** | **HL measurement** | **HL score, results** | **HL level on average** |
| --- | --- | --- | --- | --- | --- | --- | --- | --- |
| **University and college students** | | |  |  |  |  |  |  |
| Dashti et al. 2017 | Iran | Cross-sectional study | University students | N=192 | E- health literacy | eHEALS (Persian) | M: 28.21, SD: 6.95, maximum 40 | No fixed cut-off given |
| Galle et al. 2020 | Italy | Cross-sectional study | Undergraduate students | N=806 | General HL | NVS | M: 4.2, SD:1.8 (score range 0-6) | Adequate |
| Kalkbrenner et al. 2021 | USA | Cross-sectional study | Community college students | N=294 | MHL | REDFLAGS questionnaire | M: 3.93, SD: 1.11; M: 3.62, SD: 0.92* (score range 0-5) | No fixed cut-off |
| Panahi et al. 2019 | Iran | Cross-sectional study | University students | N=337 | General HL | Health Literacy for Iranian Adults Scale (HELIA) | M: 70.52, SD: 14.12 (score range 0-100) | No fixed cut-off |
| Sharma et al. 2019 | Nepal | Cross-sectional study | Undergraduate nursing students | N=152 | E- health literacy | eHEALS | Median: 3.69, IQR: 0.87 (score range 0-5), on average moderate e-HL level, score range (0-5) | Moderate |
| Tubaishat et al. 2016 | Jordan | Cross-sectional study | University students | N=541 | E- health literacy | eHEALS | M=2.62, SD:0.58 (score range: 0-5) | Moderate |
| Uysal et al. 2019 | Turkey | Cross-sectional study | Undergraduate students | N=905 participated N=1369 all students | General HL | European Health Literacy Scale | No mean given, 59% with inadequate or problematic HL | Majority (60%) inadequate |
| Vozikis et al. 2014 | Greece | Cross-sectional study | University students | N=1526 | General HL | Individual tool (see reference) | M=2.36, SD: 2.3 (score range 0-4) | Fair to high |
| **Teachers** |  |  |  |  |  |  |  |  |
| Denuwara et al. 2017 | Sri Lanka | Cross-sectional study | School teachers | N=502 | General HL | HLS-EU-Scale | No mean given, majority 61.2% (95% CI 56.9% - 65.5%) with sufficient HL | Majority (61%) sufficient |
| Yilmazel et al. 2015 | Turkey | Cross-sectional study | School teachers | N=500 | General HL | NVS | M= 2.12, SD: 1.82 (score range: 0-6) | Limited |
| **Immigrant populations** | | |  |  |  |  |  |  |
| Becera et al. 2016 | USA | Cross-sectional study | Immigrant Hispanic adults | N=3061 | General HL | Individual tool (see reference) | No mean given, majority (62%) with adequate HL | Majority (62%) adequate |
| Becera et al. 2015 | USA | Cross-sectional study | Asian Immigrants | N=4045 | General HL | Individual tool (see reference) | No mean given, majority (76%) with adequate HL | Majority adequate (76%) |
| Morris et al. 2021 | USA | Cross-sectional study | African immigrants | N=75 | General HL | NVS, Health Literacy Skills Instrument - Short Form | No mean given majority with limited HL (NVS 65% limited HL and Health literacy skill instrument 60%) | Majority (65% & 60%) limited |
| **Clinical populations** |  |  |  |  |  |  |  |  |
| Apolinario et al. 2013 | Brazil | Cross-sectional study | Hospital patients | N=322 | General HL | Test of functional HL in adults short form (S-TOFHLA (Brazilian) | No mean given, majority (68.3%) with adequate HL | Majority (68.3%) adequate |
| Blizniuk et al. 2014 | Belarus | Cross-sectional study | Hospital patients | N=281 | OHL | Oral Health Literacy Instrument (OHLI) (Russian) | M: 77.2, SD: 14.5 (score range: 0-100) | No cut-off given |
| Jamieson et al. 2013 | Australia and USA | Cross-sectional study | Indigenous Australians and American Indians (partly visiting health clinics) | N=468 (Indigenous Australians) N=254 (American Indians) | OHL | REALD-30 instrument | M: 15, SD: 7.8 and 13.7, SD: 5.3, score range (0-30) | No cut-off given |
| Jeppesen et al. 2009 | USA | Cohort study | Diabetes patients | N=225 | General HL | S-TOFHLA | No mean given, majority (84,9%) with adequate HL | Majority adequate |
| Sabbahi et al. 2009 | Canada | Cross-sectional study | Health clinic patients | N=100 | OHL | OHLI | M: 87.2, SD: 10.2 (score range: 0-100) | No cut-off given |
| Shah et al. 2010 | USA | Cross-sectional study | Health clinic patients | N=808 (adult participants) | General HL | NVS | No mean given, 51.9% with limited HL | Limited HL in 51.9% |
| Shiferaw et al. 2020 | Ethiopia | Cross-sectional study | Chronic patients | N=423 | E- health literacy | eHEALS | M: 24.6, SD: 6.4 (score range: 8-40) | No cut-off given |
| Van der Vaart et al. 2011 | Netherlands | Cross-sectional study | Patients with rhematic diseases | N=189 | E- health literacy | eHEALS | M: 28.2, SD: 5.9 (study 1) (score range: 8-40) | No cut-off given |
| Van Duong et al. 2017 | Taiwan | Cross-sectional study | Hospital patients | N=403 | General HL | HLS-SF-12 | Not reported |  |
| **General adult populations** |  |  |  |  |  |  |  |  |
| Almubark et al. 2019 | Saudi Arabia | Cross-sectional study | Residents of Saudi Arabia | N=3557 | General HL | Single‐Item Literacy Screener (Arabic) | No mean given, 54% with adequate HL | Majority (54%) adequate |
| Aygun et al. 2020 | Turkey | Cross-sectional study | Residents of Turkey | N=826 | General HL | HLS-EU; HLS-TR-Q47 (Turkish) | No mean given, majority (67%) with inadequate or limited HL. | Majority (67%) limited |
| Jeong et al. 2016 | South Korea | Cross-sectional study | South Korean adults | N=1000 | General HL | NVS (Korean) | M: 2.91, SD: 1.91 (score range:0-6) | Inadequate |
| Kayupova et al. 2017 | Kazakhstan | Cross-sectional study | Residents of Kazakhstan | N=998 | General HL | HLS-EU-Q47 | M: 34, SD: 8.6 for men, M: 33.5, SD: 9.4 (score range: 0-50) | Sufficient |
| Kuyinu et al. 2020 | Nigeria | Cross-sectional study | Residents of Lagos State | N=1831 | General HL | Brief Health Literacy Screening tool | No mean given, majority (75%) with adequate HL | Majority (75%) adequate |
| Milner et al. 2019 | Australia | Cohort study | Australian Men | N= 8362 | General HL | Health Literacy Questionnaire | M: 14.95, SD: 3.35 (score range: 5-15) | No cut-off given |
| Nadi et al. 2020 | Iran | Cross-sectional study | Residents of Iran | N=750 | General HL | Individual tool (see reference) | M: 75.2, SD: 14.6 (score range: 33-165) | No cut-off given |
| Ramlay et al. 2020 | Malaysia | Cross-sectional study | Residents of Malaysia | N=195 | OHL | OHLI (Malay) | M: 75.1, SD: 15.6 (score range: 0-100) | No cut-off given |
| Sistani et al. 2014 | Iran | Cross-sectional study | Residents of Iran | N=97 | OHL | Oral Health Literacy Adult Questionnaire (OHL-AQ) | No mean given, majority (55,7%) with inadequate or marginal HL | Majority marginal |
| Sistani et al. 2013 | Iran | Cross-sectional study | Residents of Iran | N=1030 | OHL | OHL-AQ | M: 10.5,SD: 3 (score range 1-17) | Marginal |
| Van Duong et al. 2020 | Taiwan | Cross-sectional study | Residents of Taiwan | N=1342 | General HL and e-healthy diet literacy (e-HDL) | HLS-SF-12, e-HDL | M: 32.9, SD: 5.7 (female), M: 32.1, SD: 6.1(male), (score range 0-50)  e-HDL – M:29.2, SD: 6.4 (female), M: 28.4, SD: 6.6 (male), (score range 0-50) | No cut-off given |

M: Mean, SD: Standard deviation, HL: Health literacy

# References

1. Dashti S, Peyman N, Tajfard M, Esmaeeli H. E-Health literacy of medical and health sciences university students in Mashhad, Iran in 2016: a pilot study. Electron Physician. 2017;9(3):3966-73.

2. Gallè F, Calella P, Napoli C, Liguori F, Parisi EA, Orsi GB, et al. Are Health Literacy and Lifestyle of Undergraduates Related to the Educational Field? An Italian Survey. Int J Environ Res Public Health. 2020;17(18).

3. Kalkbrenner MT, Flinn RE, Sullivan DK, Esquivel Arteaga LE. A Mental Health Literacy Approach to Supporting First-Generation Community College Student Mental Health: The REDFLAGS Model. Community College Review. 2021;49(3):243-61.

4. Panahi R, Osmani F, Sahraei M, Ramezankhani A, Rezaei M, Aghaeian N, et al. The Predictors of Health Literacy Based on the Constructs of Health Belief Model for Smoking Prevention Among University Students. Mod Care J. 2019;16(2):e87068.

5. Sharma S, Oli N, Thapa B. Electronic health-literacy skills among nursing students. Adv Med Educ Pract. 2019;10:527-32.

6. Tubaishat A, Habiballah L. eHealth literacy among undergraduate nursing students. Nurse Educ Today. 2016;42:47-52.

7. Uysal N, Ceylan E, Koç A. Health literacy level and influencing factors in university students. Health Soc Care Community. 2020;28(2):505-11.

8. Denuwara H, Gunawardena NS. Level of health literacy and factors associated with it among school teachers in an education zone in Colombo, Sri Lanka. BMC Public Health. 2017;17(1):631.

9. Yılmazel G, Cetinkaya F. Health literacy among schoolteachers in Çorum, Turkey. Eastern Mediterranean Health Journal. 2015;21:598-605.

10. Blizniuk A, Ueno M, Furukawa S, Kawaguchi Y. Evaluation of a Russian version of the oral health literacy instrument (OHLI). BMC Oral Health. 2014;14:141.

11. Sabbahi DA, Lawrence HP, Limeback H, Rootman I. Development and evaluation of an oral health literacy instrument for adults. Community Dent Oral Epidemiol. 2009;37(5):451-62.

12. Almubark R, Basyouni M, Alghanem A, Althumairi N, Alkhamis D, Alharbi LS, et al. Health literacy in Saudi Arabia: Implications for public health and healthcare access. Pharmacol Res Perspect. 2019;7(4):e00514.

13. Kayupova G, Turdaliyeva B, Tulebayev K, Van Duong T, Chang PW, Zagulova D. Health Literacy among Visitors of District Polyclinics in Almaty, Kazakhstan. Iran J Public Health. 2017;46(8):1062-70.

14. Kuyinu YA, Femi-Adebayo TT, Adebayo BI, Abdurraheem-Salami I, Odusanya OO. Health literacy: Prevalence and determinants in Lagos State, Nigeria. PLoS One. 2020;15(8):e0237813.

15. Aygun O, Cerim S. The relationship between general health behaviors and general health literacy levels in the Turkish population. Health Promot Int. 2020.

16. Jeong SH, Kim HK. Health literacy and barriers to health information seeking: A nationwide survey in South Korea. Patient Educ Couns. 2016;99(11):1880-7.

17. Sistani MM, Montazeri A, Yazdani R, Murtomaa H. New oral health literacy instrument for public health: development and pilot testing. J Investig Clin Dent. 2014;5(4):313-21.

18. Sistani MM, Yazdani R, Virtanen J, Pakdaman A, Murtomaa H. Oral health literacy and information sources among adults in Tehran, Iran. Community Dent Health. 2013;30(3):178-82.
