# Supplementary material for: Modifiable predictors of health literacy in working-age adults - a rapid review and meta-analysis
Source: BMC Public Health. 2022 Jul 30;22:1450. doi: 10.1186/s12889-022-13851-0 (PMC9338662; doi:10.1186/s12889-022-13851-0)
Supplement: Supplementary file 6 — Additional file 6. Meta-analysis and heterogeneity analysis. [file 12889_2022_13851_MOESM6_ESM.zip › Meta-analysen-heterogenita╠êtsanalysen/Internet use & HL - Meta analysis.pdf]

Results

Correlation Coefficients

Random-Effects Model (k = 9)

	Estimate	se	Z	p	CI Lower Bound	CI Upper Bound
Intercept	0.354	0.0735	4.82	<.001	0.210	0.498
	.	.	.	.	.	.

Note. Tau^2 Estimator: Restricted Maximum-Likelihood

[3]

Heterogeneity Statistics

Tau	Tau^2	I^2	H^2	R^2	df	Q	p
0.210	0.044 (SE= 0.0243)	91.93%	12.396	.	8.000	114.984	<.001

The analysis was carried out using the Fisher r-to-z transformed correlation coefficient as the outcome measure. A random-effects model was fitted to the data. The amount of heterogeneity (i.e., tau^2), was estimated using the restricted maximum-likelihood estimator (Viechtbauer 2005). In addition to the estimate of tau^2, the Q-test for heterogeneity (Cochran 1954) and the I^2 statistic are reported. In case any amount of heterogeneity is detected (i.e., tau^2 > 0, regardless of the results of the Q-test), a prediction interval for the true outcomes is also provided. Studentized residuals and Cook's distances are used to examine whether studies may be outliers and/or influential in the context of the model. Studies with a studentized residual larger than the 100 x (1 - 0.05/(2 X k))th percentile of a standard normal distribution are considered potential outliers (i.e., using a Bonferroni correction with two-sided alpha = 0.05 for k studies included in the meta-analysis). Studies with a Cook's distance larger than the median plus six times the interquartile range of the Cook's distances are considered to be influential. The rank correlation test and the regression test, using the standard error of the observed outcomes as predictor, are used to check for funnel plot asymmetry.

A total of k=9 studies were included in the analysis. The observed Fisher r-to-z transformed correlation coefficients ranged from 0.0581 to 0.7234, with the majority of estimates being positive (100%). The estimated average Fisher r-to-z transformed correlation coefficient based on the random-effects model was \hat{\mu} = 0.3540 (95% CI: 0.2099 to 0.4981). Therefore, the average outcome differed significantly from zero (z = 4.8157, p < 0.0001). According to the Q-test, the true outcomes appear to be heterogeneous (Q(8) = 114.9841, p < 0.0001, tau^2 = 0.0440, I^2 = 91.9326%). A 95% prediction interval for the true outcomes is given by -0.0817 to 0.7897. Hence, although the average outcome is estimated to be positive, in some studies the true outcome may in fact be negative. An examination of the studentized residuals revealed that none of the studies had a value larger than ± 2.7729 and hence there was no indication of outliers in the context of this model. According to the Cook's distances, none of the studies could be considered to be overly influential. Neither the rank correlation nor the regression test indicated any funnel plot asymmetry (p = 0.3454 and p = 0.3962, respectively).

Forest Plot

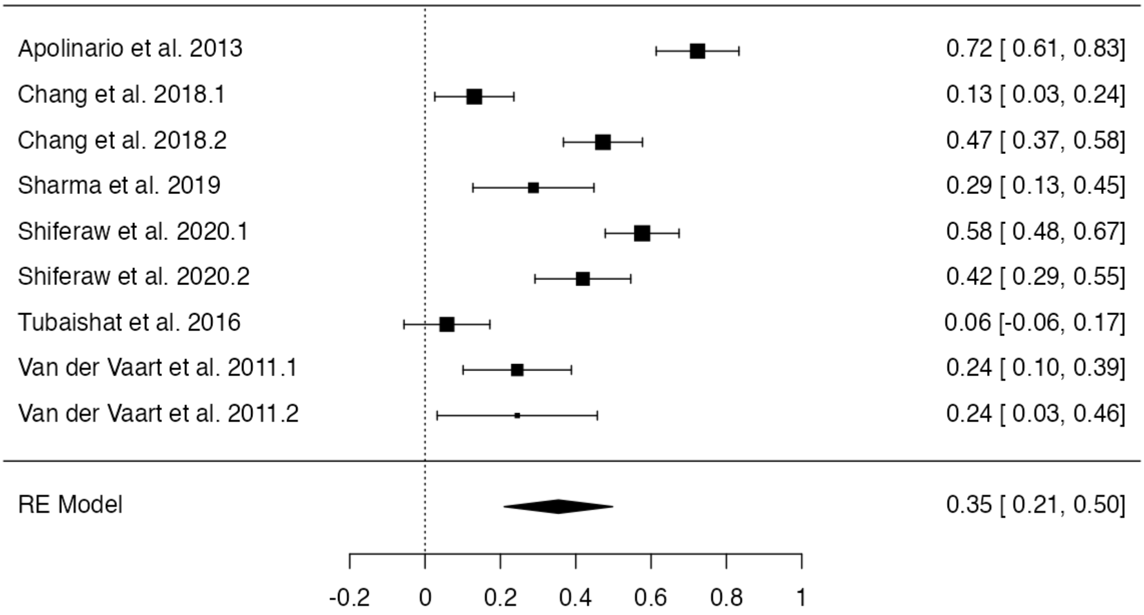

[3]

Publication Bias Assessment

Test Name	value	p
Fail-Safe N	1002.000	< .001
Begg and Mazumdar Rank Correlation	-0.254	0.345
Egger's Regression	-0.848	0.396
Trim and Fill Number of Studies	0.000	.

Note. Fail-safe N Calculation Using the Rosenthal Approach

Funnel Plot

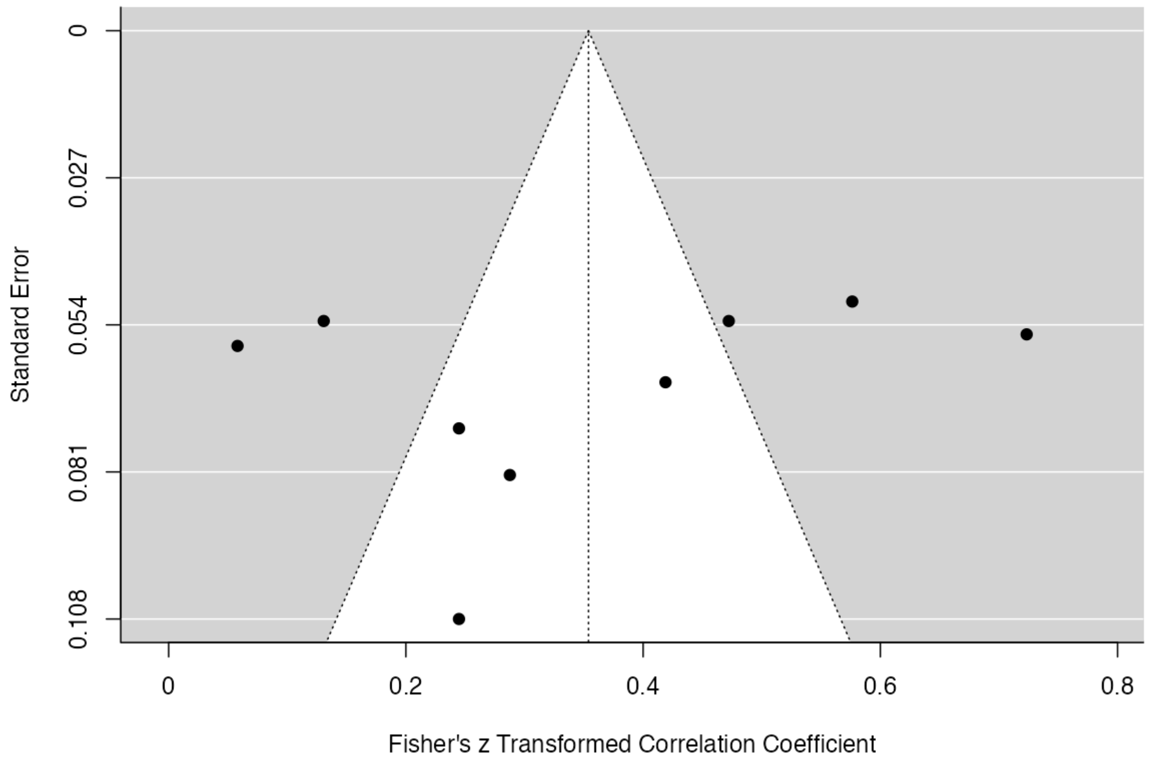

[3]

References

[1] The jamovi project (2021). *jamovi*. (Version 1.6) [Computer Software]. Retrieved from https://www.jamovi.org.

[2] R Core Team (2020). *R: A Language and environment for statistical computing*. (Version 4.0) [Computer software]. Retrieved from https://cran.r-project.org. (R packages retrieved from MRAN snapshot 2020-08-24).

[3] Viechtbauer, W. (2010). Conducting meta-analyses in R with the metafor package. *Journal of Statistical Software. link, 36*, 1-48.
